# Programmed genomic instability regulates neural transdifferentiation of human brain microvascular pericytes

**DOI:** 10.1186/s13059-021-02555-0

**Published:** 2021-12-09

**Authors:** Saba Rezaei-Lotfi, Filip Vujovic, Mary Simonian, Neil Hunter, Ramin M. Farahani

**Affiliations:** 1 IDR/Westmead Institute for Medical Research, Westmead, NSW 2145 Australia; 2 School of Medical Sciences, Faculty of Medicine and Health, University of Sydney, Sydney, NSW 2006 Australia

## Abstract

**Background:**

Transdifferentiation describes transformation in vivo of specialized cells from one lineage into another. While there is extensive literature on forced induction of lineage reprogramming in vitro, endogenous mechanisms that govern transdifferentiation remain largely unknown. The observation that human microvascular pericytes transdifferentiate into neurons provided an opportunity to explore the endogenous molecular basis for lineage reprogramming.

**Results:**

We show that abrupt destabilization of the higher-order chromatin topology that chaperones lineage memory of pericytes is driven by transient global transcriptional arrest. This leads within minutes to localized decompression of the repressed competing higher-order chromatin topology and expression of pro-neural genes. Transition to neural lineage is completed by probabilistic induction of R-loops in key myogenic loci upon re-initiation of RNA polymerase activity, leading to depletion of the myogenic transcriptome and emergence of the neurogenic transcriptome.

**Conclusions:**

These findings suggest that the global transcriptional landscape not only shapes the functional cellular identity of pericytes, but also stabilizes lineage memory by silencing the competing neural program within a repressed chromatin state.

**Supplementary Information:**

The online version contains supplementary material available at 10.1186/s13059-021-02555-0.

## Background

In a homeostatic state, the functional identity of differentiated cells is established by epigenetic mechanisms that confine the retrieval of encoded genetic information to specific “open domains” of chromatin [1, 2]. However, under experimental conditions terminally differentiated somatic cells can be reprogrammed to become pluripotent [3, 4] or to directly adopt an alternative functional identity [5, 6]. The latter phenomenon of direct cell type conversion, termed transdifferentiation (TD), can also occur naturally without exogenous interference. In *Caenorhabditis elegans*, postmitotic hindgut cells transdifferentiate into motor neurons [7] by robust and stage-wise remodelling of chromatin [8, 9]. High efficiency and reproducibility of outcome are distinguishing features of invariant integration-free [10–12] transdifferentiation [9, 13]. These features reflect, in part, the avoidance of an intermediate pluripotent state [9, 13]. Such direct conversion potentially requires robust transition from one open chromatin landscape to another while bypassing global opening of chromatin [2] that signifies the pluripotent state. Molecular regulation of this phenomenon, that involves destabilization of the original chromatin topology and coupled emergence of a “competing” chromatin topology, remains largely unknown. Given the unique features of TD, understanding the molecular governance of this phenomenon offers significant therapeutic potential in regenerative medicine.

Human microvascular pericytes demonstrate an inherent propensity for neuronal transdifferentiation [14–16]. This lineage reprogramming can be experimentally induced by forced expression of specific transcription factors [15], but it can also occur via transdifferentiation [14, 16]. At first glance, the phenomenon may not be surprising given the developmental origin of microvascular pericytes from cephalic neural crest [17], a population that exhibits neurogenic potential [18]. However, it is not obvious by what mechanism the neurogenic potential of neural crest cells is poised and then retrieved during TD of pericytes. Here, we show that competing higher-order chromatin topologies establish functional identities of pericytes and neural progenitors in a mutually exclusive manner. Accordingly, the transcriptional landscape of pericytes not only shapes the functional identity of this cell type in stable microvessels, but it also silences and protects the competing neural differentiation program by induction of a repressed higher-order chromatin state. We demonstrate that destabilization of the higher-order chromatin topology of pericytes, by stressor-mediated transient global inhibition of transcription, is sufficient for rapid emergence of the repressed neurogenic program. Completion of lineage reprogramming requires R-loop-mediated induction of DNA cleavage that remodels the smooth muscle transcriptional profile of pericytes prior to emergence of the neural transcriptome. In other words, the transcriptional profile of pericytes masks and protects the neurogenic program in a poised state that is rapidly accessed and re-activated via TD during neural regeneration [19].

## Results

### Brain microvascular pericytes reside in a metastable state, poised for neural TD

**Fig. 1:**
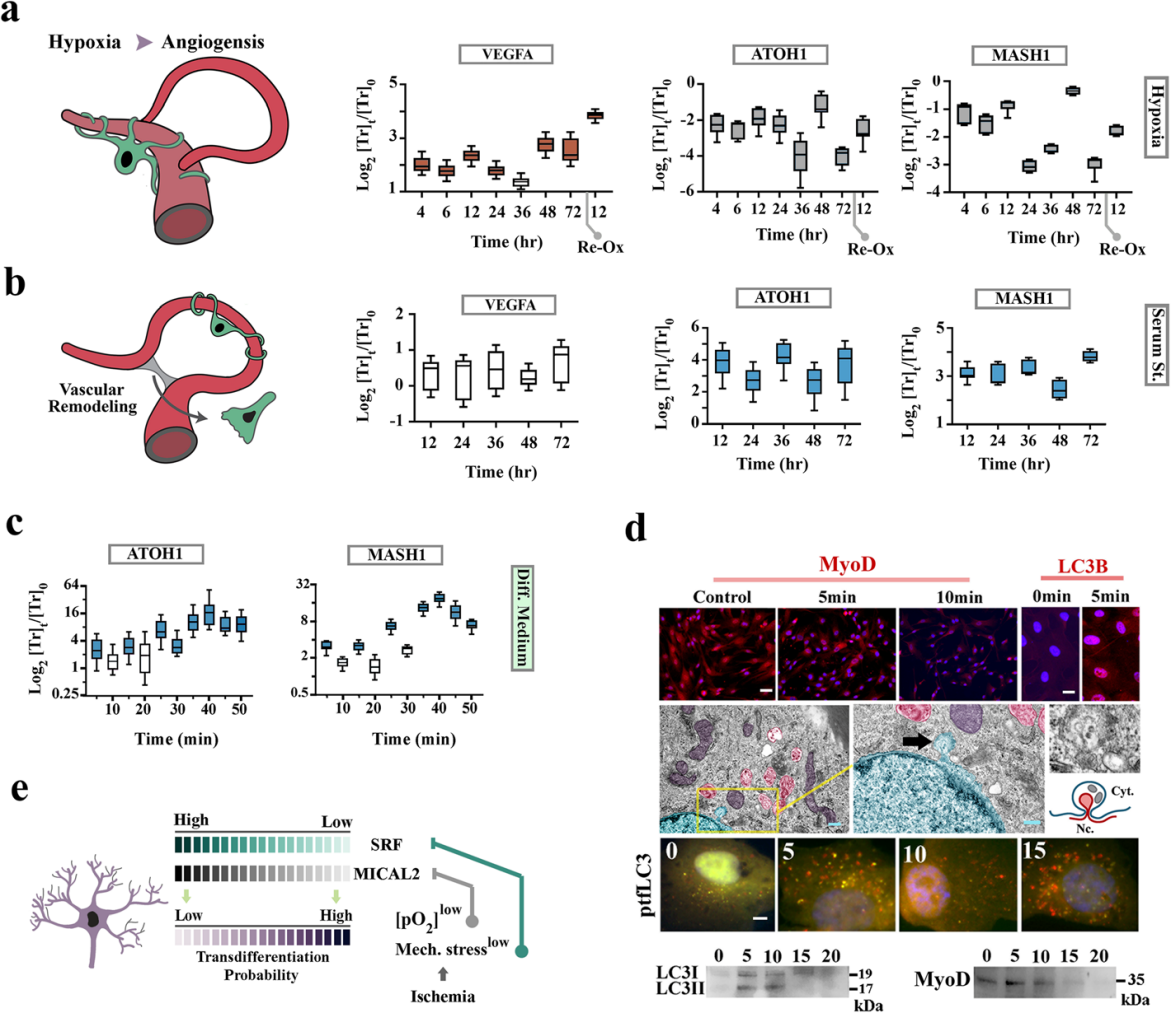
Neural TD of metastable pericytes occurs in a brief temporal window. **a** Expression of ATOH1 and MASH1, and VEGFA mRNAs at various time points after exposure of pericytes to hypoxia (coloured: *p* < 0.01). Values are normalized to normoxic control cells. **b** Expression of VEGFA, ATOH1, and MASH1 mRNAs at various time points after serum starvation (coloured: *p* < 0.01). Values are normalized to normoxic control cells in growth medium. **c** Expression of ATOH1 and MASH1 mRNAs at various time points after exposure of pericytes to N2 medium relative to control cells in growth medium (coloured: *p* < 0.01). **d** Immunohistochemical detection of MYOD (top left) and LC3B (top right). Electron micrographs show ultrastructural signature of micro-nucleophagy, an endoplasmic reticulum-derived vesicle surrounded by an autophagic vacuole (arrow). Application of an autophagy reporter plasmid (ptfLC3) confirmed that autolysosomes formed at *t* = 5 min as evidenced by detection of mRFP^low^/GFP^+^ vesicles and conversion of LC3-I to LC3-II (bottom immunoblots). Transition to mRFP^high^/GFP^−^ state and degradation of LC3-II at *t* = 15 min indicated near-complete degradation of autolysosomal contents. Scale bars top left 40 μm, top right 20 μm, middle right 0.5 μm, middle left 0.2 μm, bottom 5 μm. Immunoblots show degradation of LC3 and MyoD after initiation of transdifferentiation in pericytes (time points are shown above the blots) [85, 86]**. e** Schematic demonstration of the proposed model for combined effect of reductive stress (low pO2) and attenuated mechano-transduction on induction of pericyte transdifferentiation. Mechanical cues that sustain the activity of serum response factor (SRF) and an oxidative milieu are required for signalling by SRF co-activator, MICAL2 (microtubule-associated monooxygenase)

It is known that microvascular pericytes express neural markers including class III β-tubulin [20] and NG2 [21] in angiogenic milieu [14]. Given the well-documented coupling of neurogenesis and angiogenesis [14, 19, 22], we asked if hypoxia, as a major angiogenic stimulus, would also trigger neural TD of pericytes (Additional file 1: Fig. S1). Upon shift to a hypoxic environment, transcriptional activity of the pro-angiogenic locus, *vegfa*, increased by ≈2 fold in pericytes cultured in serum-rich (10% FBS) growth medium (Fig. 1a). We then probed the neural transdifferentiation propensity of pericytes by measuring the baseline expression of the pro-neural transcription factors, *atoh-1* and *mash-1*. To our surprise, hypoxia alone suppressed the TD propensity of pericytes by reducing the expression level of *atoh-1* and *mash-1* (*p* < 0.001, Fig. 1a). Given that angiogenesis in a mature tissue demands transient modulation of blood flow (transient hypo-perfusion) to allow for expansion of the existing microvascular bed [23, 24], we reasoned that attenuated mechano-transduction is another major angiogenic cue sensed by angiogenic pericytes in addition to hypoxia. The effect of attenuated mechano-transduction on pericytes cultured in 2D monolayers was simulated by serum withdrawal, to inhibit serum response factor [25] that operates as the master upstream regulator of the mechano-transduction pathway [25, 26]. Serum withdrawal led to a slight but insignificant downregulation of *vegfa* (two-tailed *p* > 0.05) and sustained upregulation of ATOH1 and MASH1 (two-tailed *p* < 0.01, Fig. 1b). In order to study the combined effect of hypoxia (i.e., shift to a reductive state) and hypo-perfusion on neural TD, we cultured pericytes in serum-free neurobasal medium containing the reductive N2 supplement (0.25 mM transferrin-bound Fe^3+^, redox potential of Fe^3+^/Fe^2+^: + 0.77 volts). Within 5 min of switching from growth medium to N2 medium, the pro-neural transcription factors ATOH1 and MASH1 were upregulated and by 40 min, the expression level of these transcription factors was ≈16 fold higher than control cells cultured in growth medium (two-tailed *p* < 0.01, Fig. 1c). We also noted cytoplasmic translocation of bHLH pro-myogenic transcription factor MyoD [27, 28] in transdifferentiating pericytes at *t* = 5 min followed by degradation at *t* = 10 min. Degradation of MyoD coincided with detection of nucleophagy and amplification of autophagic flux at *t* = 5 min, followed by depletion of LC3-II at *t* = 15 min (Fig. 1d). This observation suggested that amplified autophagy could be responsible for erasing myogenic memory at the protein level, a notion that was confirmed by subsequent experiments.

We concluded that sustained integration of signals generated downstream to mechano-transduction together with an oxidized milieu are essential to preserve the functional identity of pericytes as functional contractile cells. Attenuation of the latter cues during angiogenesis enhances the neural transdifferentiation propensity of pericytes (Fig. 1e). Validity of the proposed model is strengthened by reports demonstrating that the master regulator of smooth muscle differentiation, Myocardin/serum response factor complex [27, 28] requires mechanical cues [25, 26], and its co-activator, MICAL2, can only function in an oxidized cellular state [29] to sustain signalling output and prevent de-differentiation of cells. We then asked how natural TD is triggered by the paucity of the latter cues.

### Interconversion of competing higher-order chromatin topologies underpins initiation of TD

**Fig. 2:**
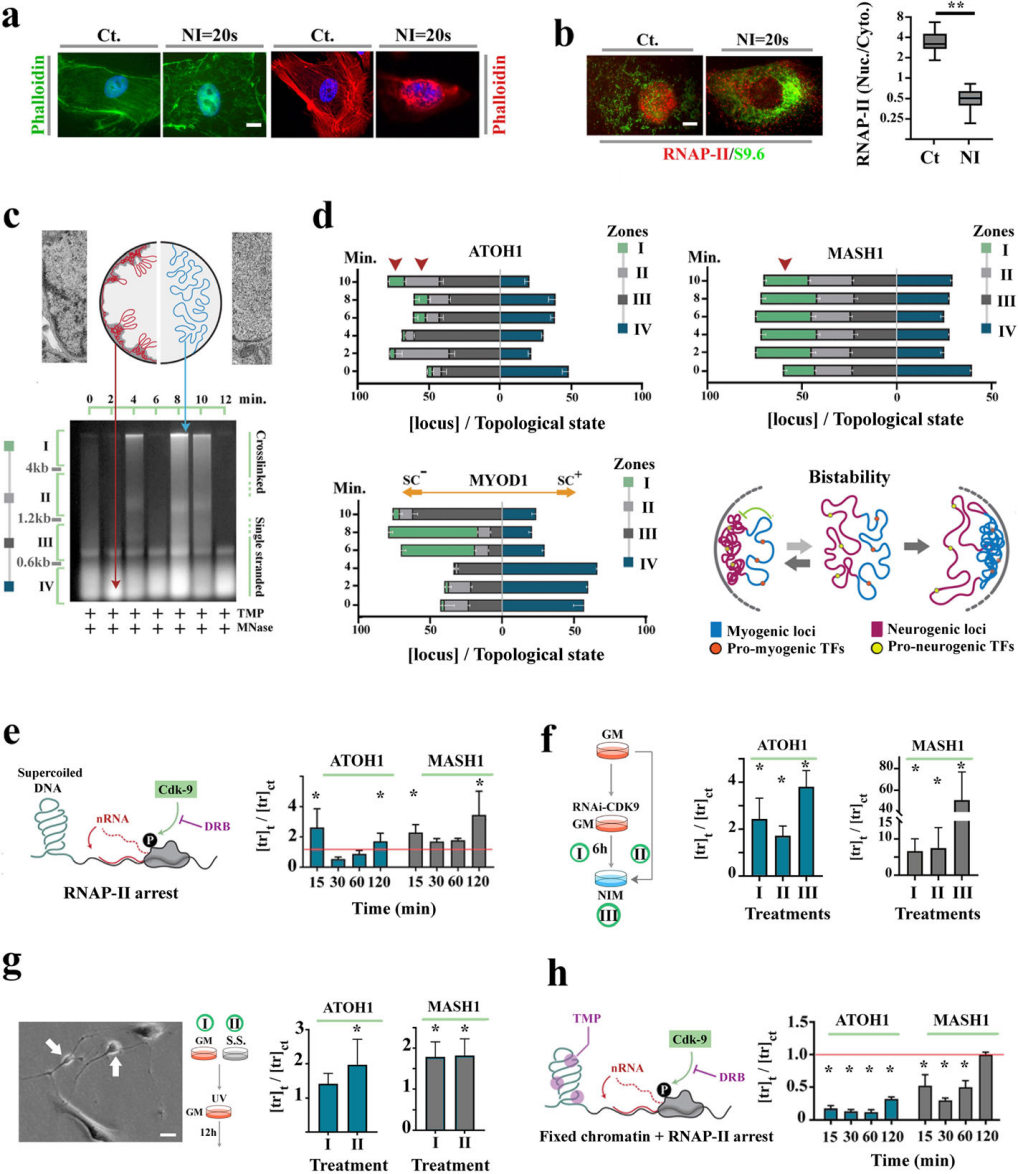
Altered higher-order chromatin topology destabilizes the lineage memory of pericytes. **a** Micrographs show appearance of nuclear Phalloidin^+^ F-actin (left: fluorescent image, right: Confocal optical slice) after 20 s of exposure to neural induction medium (NI). Scale bar 10 μm. **b** Immunohistochemical detection of RNAP-II CTD shows depletion of the nuclear complex after 20 s of exposure to neural induction medium (NI). Enhanced cytoplasmic S9.6 immunoreactivity (RNA-DNA hybrid reporter) was consistent with RNAP-II inhibition and subsequent removal. Scale bar 5 μm. Box plot shows the ratio of nuclear to cytoplasmic red signal (RNAP-II) in control and transdifferentiating pericytes (**p < 0.01). **c** Agarose gel shows oscillations of the higher-order chromatin topology from sc^+^ to sc^−^ state and vice versa at a frequency of ≈2 min. The isolated chromatin was fixed and fragmented by TMP and MNase, respectively. Chloroquine (5 μg/mL) was used as an interchelator to improve the resolution of 1D gel. DNA was extracted from zones I–IV and probed for the loci of interest using qPCR. **d** Normalized distribution of loci of interest in various topological states (zones I–IV) that form a gradient from sc^−^-dominated zone I, with low electrophoretic mobility, to sc^+^-dominated zone IV, with high electrophoretic mobility. The schematic image shows the proposed model for metastability of pericytes. In the proposed model, global transcriptional landscape not only shapes the functional identity of pericytes but it also represses the competing chromatin state that encodes neural lineage memory. **e** The graph shows expression of ATOH1 and MASH1 in DRB-treated pericytes normalized to non-treated control pericytes (culture condition: growth medium, *p < 0.01). **f** The graph shows expression of ATOH1 and MASH1 after RNAi-mediated inhibition of CDK9 in growth medium after 6 h (treatment I) compared to the level of CDK9 after neural induction (treatment II). In treatment III, cells were incubated in neural induction medium after RNAi-mediated inhibition of CDK9 (*p < 0.01). **g** The graphs show expression of ATOH1 and MASH1 after UVC radiation of cycling pericytes (treatment I) and serum-starved cells (treatment II) followed by incubation in growth medium (*p < 0.01). **h** The graph shows expression of the same genes in DRB-treated pericytes with a fixed chromatin topology (TMP-treated) normalized to DRB-treated control pericytes with native chromatin (culture condition: growth medium (*p < 0.01)

We were aware that a direct consequence of inactivation of serum response factor complex is nuclear actin polymerization [29]. Given that nuclear actin polymerization is the earliest driving event in de-differentiation of somatic nuclei transplanted into oocytes [30], we explored a similar signature in transdifferentiating pericytes. Application of phalloidin revealed that nuclear F-actin becomes detectable ≈20 s after the induction of transdifferentiation, only to be depolymerized within the next ≈40 s (Fig. 2a). Formation of F-actin was a clear indication of inactivation of serum response factor complex [29], the master regulator of smooth muscle phenotype. Could the formation of nuclear F-actin be linked to the upregulated transcription from pro-neural loci, ATOH1 and MASH1?

We found that formation of F-actin coincided with global depletion of RNA polymerase II (RNAP-II) from the nucleus (Fig. 2b). This finding was not surprising as monomeric actin becomes inaccessible within the polymerized form, leading to impaired RNAP-II processivity [31] and eventual degradation of the stalled enzymatic complex [32]. However, it remained unclear how the global arrest of RNAP-II activity after the formation of F-actin anticipates upregulated transcription from ATOH1 and MASH1 loci*.* A plausible explanation was that in the absence of rotational torque generated by RNAP-II [33, 34], higher-order chromatin organization may change [35] leading to resolution of topological constraints that restrict transcription of pro-neural genes [36]. To test the validity of this rationale, it was necessary to study higher-order chromatin topology [37] of the pro-neural loci in transdifferentiating cells. Pericytes undergoing transdifferentiation in N2 medium were lysed at 2-min intervals, and 4,5′,8-trimethylpsoralen (TMP)/UV-A was employed to induce DNA inter-strand crosslinks [37] in order to stabilize chromatin topology prior to isolation and fragmentation of DNA (Fig. 2c). Next, DNA was purified based on electrophoretic mobility, from four defined zones (Fig. 2c). DNA with lower mobility in zone I is hyper-negatively supercoiled and gradually transitions to a hypermobile positively supercoiled topology in zone IV (Fig. 2d). Finally, qPCR was utilized to quantify the distribution of pro-neural loci ATOH1 and MASH1, and the pro-myogenic locus *myod1* in four genomic fractions corresponding to zones I–IV of Fig. 2c.

Within 2 min of transdifferentiation, ATOH1 loci adopted a negatively supercoiled (sc^−^) configuration (≈6 fold expansion of zone II in Fig. 2d, two-tailed *p* < 0.0001). Also, MASH1 loci with sc^−^ topology in zone I increased by ≈1.8 fold from *t* = 0 to *t* = 2 min (two-tailed *p* < 0.0001). Concurrently, we noted near-complete depletion of MYOD1 loci with sc^−^ topology in zones I and II from *t* = 0 to *t* = 4 min (two-tailed *p* < 0.0001, Fig. 2d). Notably, the observed trend was reversed from *t* = 6 to *t* = 8 min and while *MYOD1* loci adopted a sc^−^ topology as evidenced by expansion of zone I, some ATOH1 and MASH1 loci adopted a sc^+^ topology (note the expansion of zone IV, Fig. 2d). The observed oscillation of chromatin topology suggested that pericytes reside in a metastable state wherein pro-myogenic and pro-neurogenic loci compete for expression, but where transcription of pro-myogenic loci renders pro-neurogenic loci less accessible to RNAP-II [38, 39] by induction of a positively supercoiled repressed chromatin topology (Fig. 2d). In this model, F-actin-mediated depletion of RNAP-II and the resultant remodelling of higher-order chromatin topology of metastable pericytes is sufficient to trigger initial expression of pro-neural genes. We sought experimental proof for regulation of pro-neural genes by higher-order chromatin topology.

**Fig. 3:**
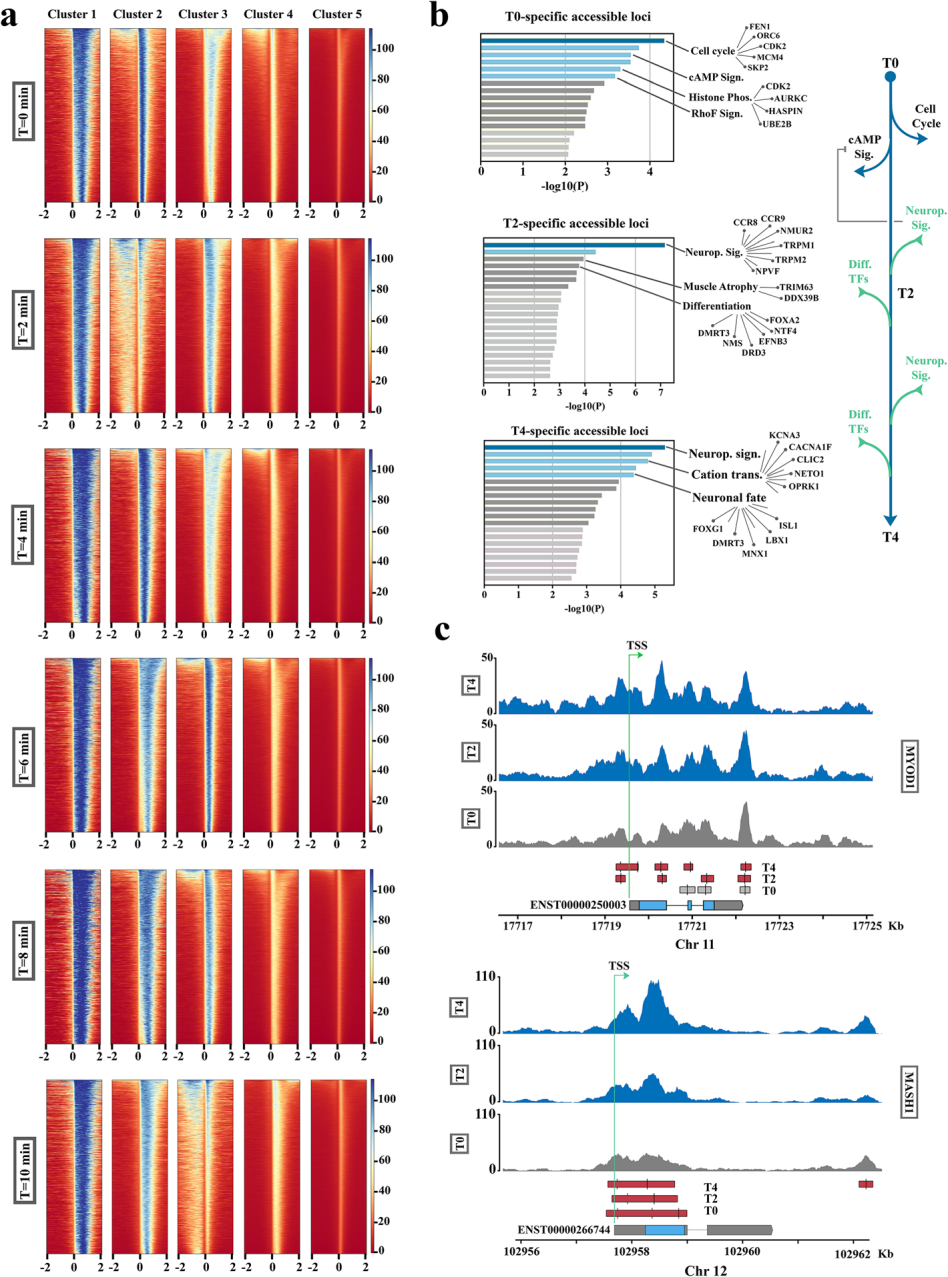
ATAC-seq analysis of genome-wide chromatin accessibility upon neural transdifferentiation of pericytes. **a** Heatmaps show density of mapped ATAC-seq reads 2 kb up- and downstream of ENSEMBL-annotated transcriptional start sites (TSS) in GRCh38 (hg38). For every time point (*n* = 6 time points), data were partitioned into 5 clusters based on *k*-means algorithm. **b** Graphs summarize GO enrichment analysis of ATAC-seq profiles of pericytes upon transdifferentiation (Analysis platform: Metascape). The top graph shows functional clustering of genomic loci that become inaccessible soon after induction of transdifferentiation, at *t* = 2 min. The middle and bottom graphs show inaccessible genomic loci in pericytes that become accessible at *t* = 2 and 4 min after induction of transdifferentiation. **c** ATAC-seq peaks demonstrate accessibility of genomic loci encoding MYOD1 and MASH1 loci at baseline and at *t* = 2 and 4 min post-induction of transdifferentiation

In order to simulate F-actin-mediated suppression of RNAP-II and global transcriptional arrest, we required a compound with rapid onset of activity that inhibited RNAP-II in a reversible manner [40]. Cyclin-dependent kinase-9 (CDK-9) is a key component of the positive transcription elongation factor (P-TEFb). Inhibition of CDK-9 by application of 100 μM DRB [41] leads to dephosphorylation of CTD of RNAP-II and premature arrest of RNA polymerase II, replicating global arrest of RNAP-II during Phase I of TD. Pharmacological inhibition of CDK9 was sufficient to upregulate expression of pro-neural genes *atoh1* and *mash1* in DRB-treated pericytes in growth medium relative to control pericytes in the same medium (Fig. 2e). This effect was prominent at 2 h, a timepoint which accommodates the maximum effect of DRB. We next employed a cocktail of two siRNAs to target CDK9 (Fig. 2f). After electroporation, cells were incubated in growth medium for 6 h. The level of ATOH1 and MASH1 transcripts in CDK9^RNAi^ cells was comparable to that of transdifferentiating pericytes in neural induction medium (Fig. 2f). Notably, combination of RNAi-mediated inhibition of CDK9 and exposure to neural induction medium causes a synergistic amplification of transcription from ATOH1 and MASH1 loci relative to control transdifferentiating cells (Fig. 2g). Finally, we utilized UV irradiation (se methods) to induce RNAP-II stalling [42]. The application of UV irradiation followed by incubation in growth medium for 12 h enhanced transcription from ATOH1 and MASH1 pro-neural loci. Notably, stabilization of the negatively supercoiled chromatin domains in pericytes by application of TMP/UV-A, prior to application of DRB, prevented upregulation of ATOH1 and MASH1 in [TMP + DRB]-treated cells compared to DRB-treated cells (Fig. 2h). It was concluded that F-actin-mediated depletion of RNAP-II in pericytes of angiogenic vessels leads to local relaxation (de-repression) of higher-order chromatin topology and upregulation of pro-neural genes during early TD (Phase I of TD). Therefore, transdifferentiating pericytes transition from the metastable state into a bistable transcriptional state characterized by co-expression of pro-neural and pro-myogenic transcription factors. To confirm this finding at a global level, we applied ATAC-seq and mapped the chromatin accessibility of transdifferentiating pericytes (Fig. 3a). Analysis of ATAC-seq, based on *k*-means algorithm, revealed an abrupt global shift of chromatin accessibility relative to ENSEMBL-annotated transcription start site (TSS), in a major fraction of active genomic loci (cluster 2) at *t* = 2 min after the induction of transdifferentiation (Fig. 3a). Subsequent GO enrichment analysis of ATAC-seq results showed that a genic cluster encoding cell cycle-related genes and cAMP signalling pathway components becomes inaccessible at *t* = 2 min after transdifferentiation. In contrast, genomic loci encoding neuropeptide signalling (a major antagonistic pathway of cAMP signalling) and neural fate determination transcription factors become accessible after *t* = 2 and 4 min post-induction of transdifferentiation (Fig. 3b). Also corroborating the findings from investigation of locus topology, we noted that the genomic loci encoding MYOD1 and MASH1 show enhanced chromatin accessibility subsequent to TD (more prominent in MASH1) at *t* = 2, 4 min (Fig. 3c). The latter finding was consistent with suggestion that upon induction of TD pericytes transition into a state of bistability characterized by competition between pro-myogenic and pro-neurogenic fates. We then focused on the mechanism of progression from bistability to neural commitment, a phenomenon that requires obliteration of smooth muscle transcriptional memory (Phase II of TD).

### Induction of R-loops is essential for resolution of bistability and completion of lineage reprogramming

**Fig. 4:**
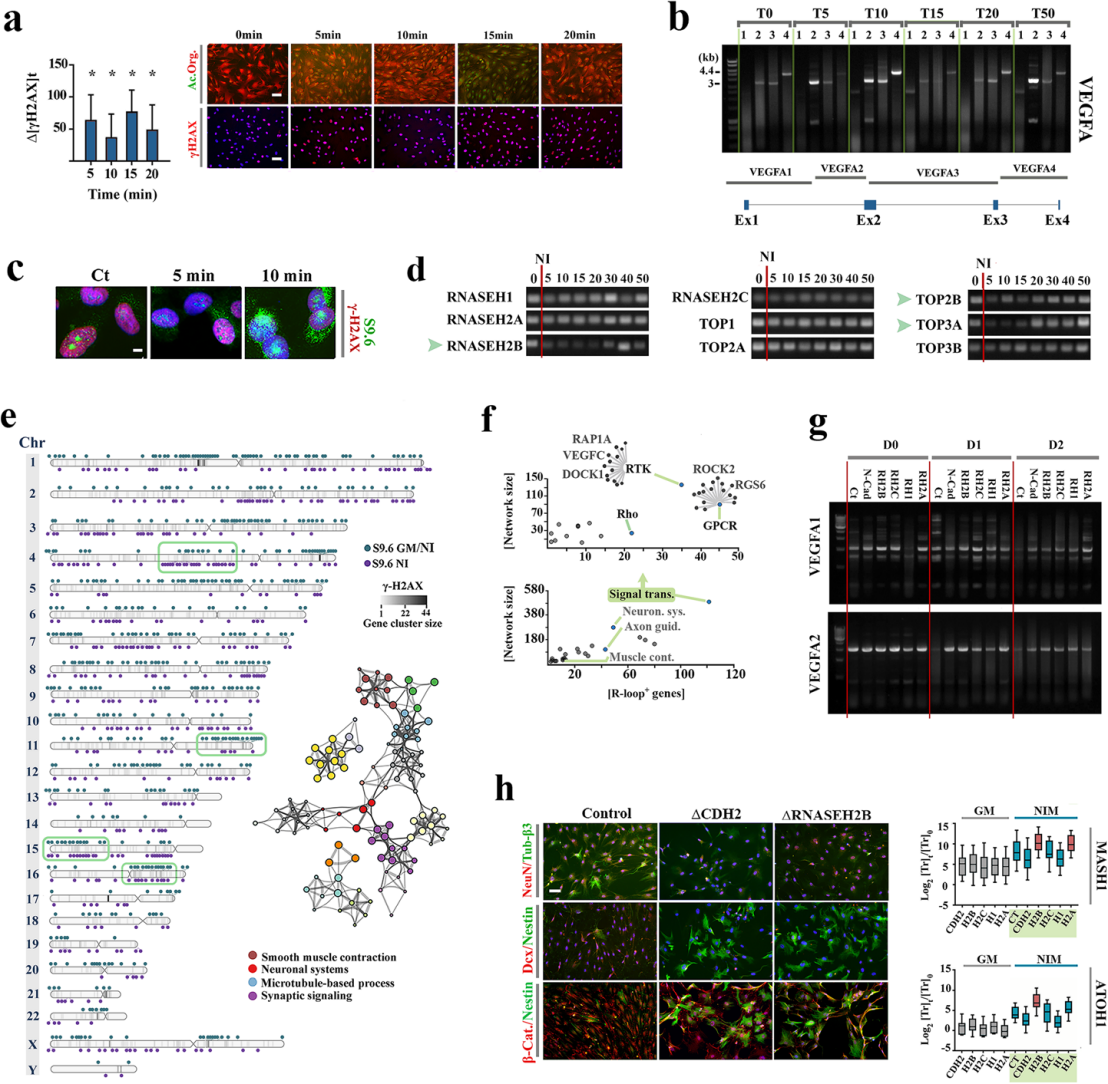
Induction of R-loops drives transition of bistable Phase I transdifferentiating pericytes to monostable Phase II neural progenitors. **a** Quantification of γH2AX immunofluorescence signal in cultured pericytes after induction of transdifferentiation (*y*-axis: increased intensity (*∆*) of the fluorescent signal in transdifferentiating cells relative to control cells, *p < 0.01). Acridine orange staining shows depletion of RNA concurrent with induction of DNA damage at *t* = 15 min. **b** Gel shows DNA integrity of *vegfa* locus at various timepoints (*T*: 0–50 min) during transdifferentiation of pericytes, probed using four specific PCR primers (1–4: primers as per bottom schematic figure, see Additional file 1: Table S1). **c** Micrographs show immunohistochemical detection of RNA:DNA hybrids (R-loops), using S9.6 antibody, during transdifferentiation of pericytes. Scale bar 10 μm. **d** Transcriptional fingerprinting of RNASEH1, RNASEH2 subunits, and topoisomerase subunits in transdifferentiating pericytes (*t* = 0–50 min) [87]. **e** DRIP-seq profile of transdifferentiating pericytes shows chromosomal distribution of R-loops 10 min after induction of transdifferentiation. turquoise: R-loop^+^ loci shared by control cells in growth medium (GM) and transdifferentiating cells in neural induction medium (NI), purple: R-loop^+^ loci specific to transdifferentiating cells. Greyscale heatmap in the chromosome body shows γH2AX ChIP-seq profile of transdifferentiating cell. GO enrichment analysis (right network) revealed that the R-loop^+^ genes are predominantly involved in regulating smooth muscle vs. neuronal physiology. **f** The impact of R-loop formation on rewiring the existing functional network topology was predicted by analyzing the DRIP-seq profile of transdifferentiating pericytes. The bottom graph shows the number of R-loop^+^ genes in functional GO clusters (*x*-axis) plotted against the total number of network connections established by the R-loop^+^ genes in the cluster (*y*-axis). Signal transduction pathways accommodate the highest number of R-loop^+^ genes with extensive connectivity profile. Further analysis of the signalling pathways based on the same approach revealed that G-protein coupled receptor, Rho, and receptor tyrosine kinase signalling cascades are more likely to be disrupted by induction of R-loops during transdifferentiation of pericytes (top graph). **g** Gels demonstrate the impact of forced expression of N-cadherin, RNASEH1, and RNAHEH2 subunits on R-loop-mediated DNA cleavage that affects vegfa locus (**b**) upon induction of pericyte transdifferentiation (D: days post-induction). Note that amplified activity of RNaseH and N-cadherin prevents transdifferentiation-mediated cleavage of VEGF-1 and VEGFA-2. **h** Immunohistochemical profile of transdifferentiating pericytes (3 days post-induction) shows immature Nestin^−^/Dcx^+^ neurons and fewer β3-tubulin^+^ neurons (refer to Additional file 1: Fig. S4 for quantification). Forced expression of N-cadherin (ΔCDH2) and RNASEH2B (ΔRNASEH2B) blocks transdifferentiation evidenced by presence of Nestin^+^/Dcx^−^ population. Expression of ATOH1 and MASH1 upon overexpression of CDH2 and RNAHSEH subunits in pericytes cultured in growth medium (GM: grey) and after neural induction (*t* = 24 h) (Red: *p* < 0.01). Scale bar 40 μm

Insight into neural commitment was afforded by the Rad-51^+^/γH2AX^+^ profile of transdifferentiating pericytes (Fig. 4a) suggesting that DNA cleavage [43, 44] is a consequence of Phase I events. Intense γH2AX immunoreactivity was detected at *t* = [5, 15] minutes after induction of transdifferentiation (Fig. 4a). Probing the chromatin integrity of *vegfa* locus a highly transcribed gene in differentiated pericytes [45], confirmed that DNA cleavage interrupted the locus particularly at *t* = [5, 15] minutes (Fig. 4b). We reasoned that enrichment of hyper-negatively supercoiled chromatin at *t* = [4, 10] minutes (Fig. 2c) could provide a mechanistic explanation for occurrence of DNA cleavage at *t* = [5, 15] minutes. A corollary of adopting a negatively supercoiled state is that annealing of nascent RNA to the underwound (sc^−^) DNA is facilitated which leads to induction of RNA:DNA hybrids in GC-rich genomic regions, unprotected looping of the non-template strand of DNA [46], and occurrence of DNA double-stranded breaks [47].

Application of S9.6 antibody to detect RNA:DNA hybrids disclosed extensive formation of R-loops at *t* = 10 min post-induction of transdifferentiation (Fig. 4c), following global enrichment of hyper-negatively supercoiled DNA that occurs at *t* = 6–8 min. Induction of R-loops is a deterministic phenomenon that affects the entire population as > 95% of S9.6^+^ pericytes become non-responsive to mitogenic cues within 10 min of transdifferentiation (Additional file 1: Fig. S2). Consistent with the S9.6^+^ profile of pericytes, we found that RNASEH2 subunits and in particular, RNASEH2B, rapidly decline upon transdifferentiation of pericytes (Fig. 4d). RNASEH2 is essential for hydrolysis of RNA:DNA hybrids and its H2B subunit enhances processivity of the enzyme complex by an order of magnitude [48]. Another feature of transdifferentiation in pericytes was that Rad-51 was suppressed in these cells (Additional file 1: Fig. S3). Inhibition of Rad-51 prevents induction of *trans* R-loops while co-transcriptional induction of R-loops (in *cis*) remains unaffected [49]. Accordingly, R-loops would preferentially affect loci that are transcriptionally active in transdifferentiating pericytes and potentially contribute to elimination of the smooth muscle identity. We, therefore, hypothesized that R-loop-mediated DNA cleavage may facilitate transition from bistability to neural commitment during Phase II of TD by preferential disruption of smooth muscle transcriptional landscape. To validate our hypothesis, we probed the genome-wide distribution of R-loops and DNA cleavage in transdifferentiating pericytes (*t* = 10 min) using DRIP-seq (DNA-RNA immunoprecipitation) and γH2AX ChIP-seq, respectively (Fig. 4e).

Amongst the loci identified as R-loop^+^, 56% were shared by the control and transdifferentiating cells and the remaining loci were specific to the transdifferentiating cells (Fig. 4e). Universal depletion of RNAP-II in the early stage of TD rules out the possibility that R-loops were carried over from the pre-transdifferentiation period. An alternative interpretation was that such commonality could arise by induction of R-loops upon recovery of smooth muscle transcriptome. Enrichment of genes that regulate smooth muscle differentiation, including members of G-protein signalling cascades, amongst R-loop^+^ loci, corroborated the latter notion (Fig. 4f). Signalling by G-protein coupled receptors and the downstream mediator RhoA is essential for activity of serum response factor and acquirement of smooth muscle phenotype [50]. Analysis of γH2AX ChIP-seq revealed that the majority of R-loop^+^ loci (≈51%) were simultaneously γH2AX^+^ indicating that R-loops were associated with DNA damage (Fig. 4e). Findings suggested that R-loop-mediated depletion of the smooth muscle transcriptome (Fig. 4f) could complement autophagy-mediated depletion of the smooth muscle proteomic memory (Fig. 1d) and potentially facilitate emergence of the neural transcriptome from the bistable transcriptional landscape of transdifferentiating Phase I pericytes. To validate this proposal, we investigated whether amplification of R-loop surveillance mechanisms, including RNASEH enzymes, could block transition to neural commitment (Phase II).

We interrogated the importance of R-loops in neural commitment by forced expression of RNASEH2A, RNASEH2B, RNASEH2C, and RNASEH1. Further, we employed overexpression of N-cadherin (CDH2) to stabilize the cytoplasmic actin cytoskeleton and to prevent formation of nuclear F-actin in order to block the early stage of TD. Amplified activity of N-cadherin, RNASEH2A, RNASEH2B, RNASEH2C, and RNASEH1 prevented R-loop-induced DNA cleavage in VEGFA locus (a genomic signature of transition to neural commitment during Phase II of TD) at day 1 and 2 post-induction of TD (Fig. 4g). We then asked if inhibition of DNA cleavage by amplification of RNASEH2B and N-cadherin is sufficient to effectively block TD. We employed Nestin immunoreactivity to identify cells which become Nestin^−^ upon committing to differentiation and loss of stemness [51]. NeuN and β3-tubulin (Tuj-1), on the other hand, were used as specific markers of mature [52] and immature neurons [53], respectively. Accordingly, Nestin^+^/NeuN^+^ immunoreactivity characterized reprogrammed differentiated cells as opposed to Nestin^+^/NeuN^−^ immunoreactivity of original pericytes. Absence of Doublecortin (Dcx) and β3-tubulin despite high levels of ATOH1 and MASH1 transcripts indicated that ΔRNASEH2B^high^ cells progress to Phase I (bistable phase) but do not complete Phase II of TD (Fig. 4h). On the other hand, the majority of ΔCDH2^high^ cells were characterized as NeuN^−^/β3-tubulin^−^/Dcx^−^ and the level of ATOH1 and MASH1 in these cells remained lower than the control transdifferentiating cells (Fig. 3h). This was consistent with a Phase I block (complete suppression of TD). The evidence that R-loop-induced DNA damage is critical for transition from bistability to neural commitment is in accord with a recent study demonstrating the critical role of genome-wide induction of R-loops in cell reprogramming [54]. We next questioned how dilution of the smooth muscle transcriptional profile by this mechanism leads to emergence of the neural transcriptome.

**Fig. 5:**
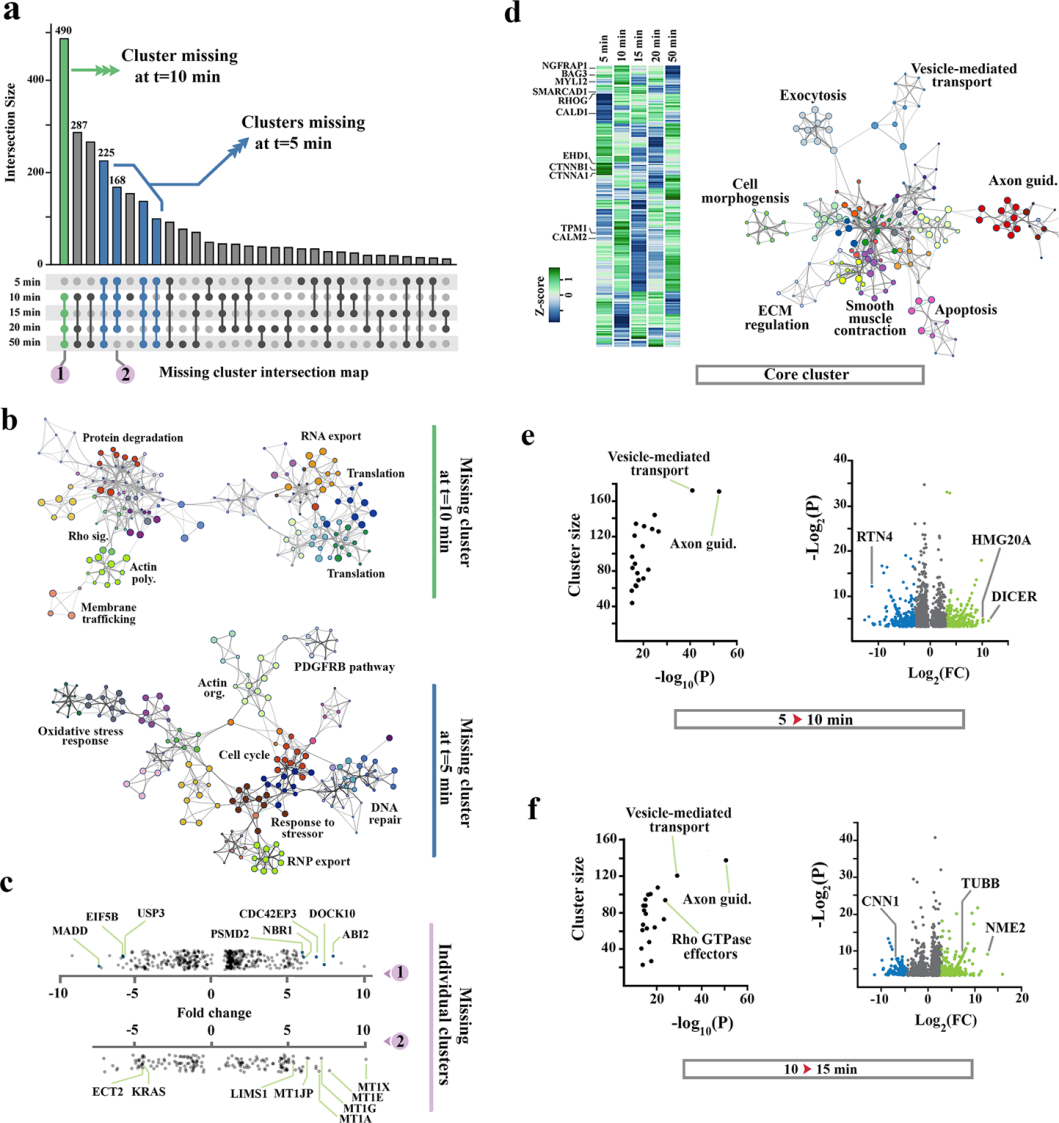
RNA-seq analysis of transcriptional remodelling upon neural transdifferentiation of pericytes. **a** Missing transcript intersection map was used to visualize temporal pattern of transcript loss. Bar plots show the size of missing clusters (filled dots: missing) for each combination of time points (*n* = 30 combinations). Note the rapid decline of transcriptomic complexity at *t* = 5 and 10 min followed by gradual recovery of the transcriptome after *t* = 20 min. **b** GO enrichment analysis of transcripts that became undetectable after *t* = 5 min (blue bars) and *t* = 10 min (green bar) revealed strong clustering in related pathways. **c** Scatter plots show RNA-seq profiles of two genic clusters corresponding to clusters 1 (green bar) and 2 (blue bars) of the missingness graph (part a). The top scatter graph shows the expression level of genic cluster 1 at *t* = 5 min (prior to missing at *t* ≥ 10 min) as normalized to control pericytes. These genes are linked to stabilization of cytoplasmic actin cytoskeleton (CDC42EP3, DOCK10) or protein translation machinery (EIF5B). The bottom scatter graph shows the expression level of genic cluster 2 at *t* = 50 min (after recovery) as normalized to control pericytes. Upregulation of these genes (e.g., Metallothionein) could facilitate tolerance of stressors by transdifferentiating pericytes. **d** Heat map shows normalized expression of genes whose transcription does not cease during transdifferentiation. GO enrichment analysis of this cluster shows evidence of emergence of a transcriptional profile compatible with neuronal functionality. **e** Scatter plots show RNA-seq profiles of transdifferentiating pericytes at *t* = 10 min normalized to transdifferentiating cells at *t* = 5 min. The left scatter plot shows Log_10_ (*p* value) of differentially regulated functional clusters plotted against the cluster size, and the right graph shows Log_2_ (fold change) of differentially expressed genes plotted against − Log_2_ (*p* value). **f** Scatter plots show RNA-seq profiles of transdifferentiating pericytes at *t* = 15 min normalized to transdifferentiating cells at *t* = 10 min

RNA-seq analysis of transdifferentiating pericytes revealed rapid decline of transcriptomic complexity at *t* = 5 and 10 min followed by gradual recovery of the transcriptome after *t* = 20 min (Fig. 5a). By comparing the remodelling transcriptome of transdifferentiating pericytes to control cells, we were able to dissect three distinct transcriptional clusters: Cluster-I comprising depletion of transcripts with smooth muscle functionality, Cluster-II encompassing a core of retained pericytic transcripts, and Cluster-III, a cluster of de novo neuronal transcripts. Upon detailed analysis of missing transcripts (C.I), we noted rapid (*t* = 5 min) depletion of transcripts whose protein products regulate the PDGFRβ signalling pathway that is pivotal for recruitment of pericytes to microvasculature and induction of smooth muscle phenotype [55] (Fig. 5b). In the same timeframe, transcripts of cytoskeletal proteins and those that regulate response to oxidative stress became undetectable. The latter transdifferentiation profile reflected the response to serum withdrawal combined with the shift to a reductive state (Fig. 5b, c). In subsequent transition to the climax of bistability (*t* = 5 min to 10 min), transcripts required for translation, protein degradation, and RNA export machinery and also transcripts that belong to Rho signalling cascade and cytoskeletal remodelling were preferentially depleted (Fig. 5b), corroborating the DRIP-Seq profile (Fig. 4e, f). Analysis of Cluster-II (a core of retained pericytic traits) revealed that depletion of Cluster-I transcripts was sufficient for rapid partial emergence of the neuronal transcriptome at *t* = 5 min (Fig. 5d). In other words, Cluster-II encompasses a fractional signature of the pericytic transcriptome that can be recruited and re-organized to facilitate emergence of neuron-specific functions, subsequent to deletion of Cluster-I transcripts. Ultimately, analysis of Cluster-III (transcripts specific to transdifferentiating pericytes) revealed that transcripts implicated in axon guidance and vesicle-mediated transport (as a precursor of synaptic transmission) were amplified by de novo expression at *t* = 10 min (Fig. 5e). This was combined with significant upregulation of generic and neuron-specific inducers of differentiation, Dicer [56] and HMG20A [57], respectively (Fig. 5e). In transition to Phase II (*t* = 15 min), we noted enhanced transcription from neuron-specific loci including TUBB (Tubulin-β1) and upregulation of the negative modulator of the Rho GTPase, NME2 [58] (Fig. 5f). Given recovery of the Rho signalling cascade at *t* = 15 min (Fig. 5f), upregulation of NME2 could inhibit Rho-mediated stress fibre formation and facilitate assembly of the neuronal growth cone by other members of the Rho signalling cascade [59]. Findings suggested that depletion of Cluster-I transcripts (pericyte-specific transcripts) is sufficient not only to drive expression of pro-neural genes (by altering localized higher-order topology of chromatin) but also to reveal the core Cluster-II transcripts that are compatible with neural identity. It became apparent that while de novo expression of neuron-specific genes contributes to the emerging transcriptional profile of transdifferentiating pericytes, a major fraction of the existing pericytic transcriptome is also recruited and re-organized to facilitate emergence of neuron-specific functions during lineage reprogramming. We then tested our final postulate that while transition to bistability (Phase I) is abrupt and deterministic, incomplete progression to monostability (neural commitment: Phase II) reflects inherent spatial and temporal stochasticity of R-loop formation in vitro*.* To test this hypothesis, we employed a variation of Cas9-mediated RNA-guided DNA targeting [60] to program the genomic distribution of DNA cleavage sites and to synchronize the induction of breaks.

**Fig. 6:**
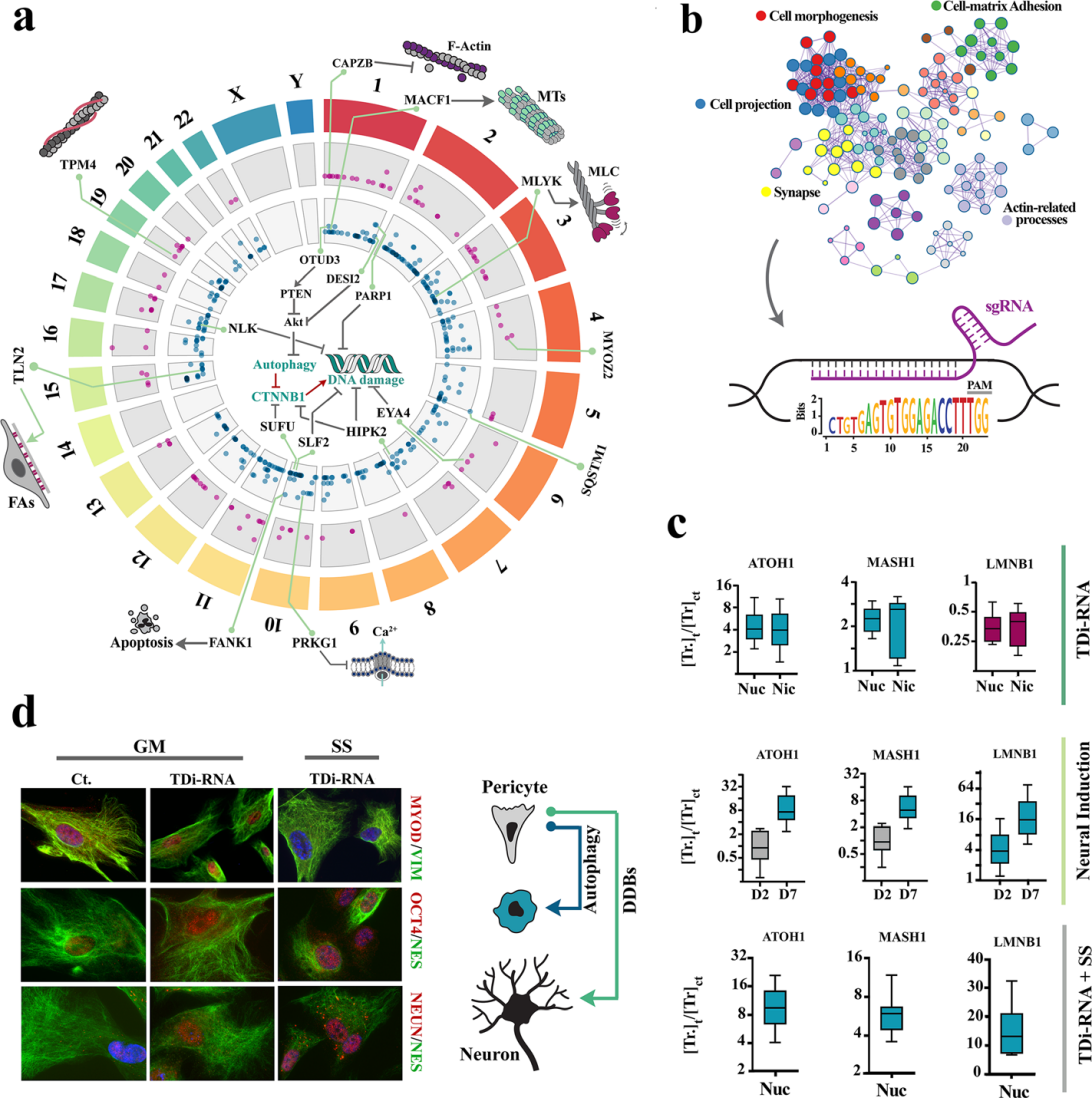
Targeted genome-wide application of CRISPR-Cas9 replicates transdifferentiation dynamics. **a** Chromosomal distribution of genes that contain a region of partial homology. Genes in the inner circle (green) do not have a flanking PAM sequence, while those in the outer circle (CRISPR target genes) do. *Y*-axis corresponds to the length of homologous seed region. **b** GO enrichment analysis (top) of the genes with a consensus CRISPR recognition motif (bottom sequence logo) revealed their involvement in cell-matrix interaction and regulation of cytoskeleton. **c** The top plots show expression of selected genes 12 h after application of TDi-RNA (Nuc: Cas9 nuclease, Nic: Cas9 Nickase). The middle plots show expression of the same genes 2 (D2) and 7 days (D7) after neural induction in control pericytes. The bottom plots show expression of selected genes 12 h after application of TDi-RNA (Cas9 nuclease) and in incubation a serum-free medium. Magenta: downregulation with *p* < 0.05, turquoise: upregulation with *p* < 0.05, grey: non-significant. **d** Panel shows immunohistochemical identification of three selected proteins before (Ct.) and after application of TDi-RNA and incubation of programmed cells in growth medium (GM) or in serum-starved condition (SS). A model is proposed whereby autophagy and double-stranded DNA breaks (DDBs) act synergistically to propel de-differentiation and subsequent neural differentiation during the transdifferentiation of pericytes

Although specificity of targeting in the CRISPR-Cas9 system is determined by the first ∼20 nucleotides of the single-guide RNA (sgRNA) [61], a shorter seed region of ∼8–13 nucleotides flanking the NGG sequence of protospacer adjacent motif (PAM) is sufficient for induction of cleavage by Cas9 [60, 62–64]. We capitalized on this property to design a highly specific sgRNA, termed transdifferentiation-inducing sgRNA (TDi-RNA), that directs Cas-9 to seed regions distributed non-randomly in a genic cluster (Fig. 6a, Additional file 1: Table S2). The genic cluster (*n* = 469 genes) was identified (Fig. 6a) based on the principle that conserved homologous regions longer than 13 bp are unlikely to be randomly seeded into the genome [65]. Gene ontology analysis revealed that members of the identified cluster were involved in regulation of focal adhesion and stabilization of actomyosin (Fig. 6b). After introduction of the TDi-RNA (sgRNA) and Cas9 nuclease or nickase, pericytes were returned to growth medium and incubated for an additional 12 h. Expression of ATOH1 and MASH1 in pericytes programmed by application of TDi-RNA increased significantly (Fig. 6c). Similar to endogenous transdifferentiation and neural induction, application of TDi-RNA decreased the expression level of Rad51. However, programmed pericytes remained bistable as evidenced by the MyoD^+^/NeuN^+^ profile of these cells (Fig. 6d). We attributed the bistability to absence of nucleophagy induced by F-actin-mediated RNAP-II arrest (Phase I of TD, Fig. 1d). Notably, induction of autophagy by incubation of the programmed cells in serum-starved condition was sufficient to erase the myogenic lineage memory of these cells (Fig. 6d). Findings supported the interpretation that inherent spatial and temporal stochasticity of R-loop formation is the key limiting factor in neural transdifferentiation of pericytes. The application of TDi-RNA also confirmed previous findings that disruption of the myogenic program is sufficient to de-repress the neurogenic program that is suppressed by global transcriptional dynamics of pericytes. One may argue that temporal stochasticity of R-loop formation acts as the key limiting step in neural transdifferentiation of brain pericytes in order to prevent rapid depletion of mural pericytes during angiogenesis.

### TD propensity of pericytes is programmed by prior exposure to stressors

**Fig. 7:**
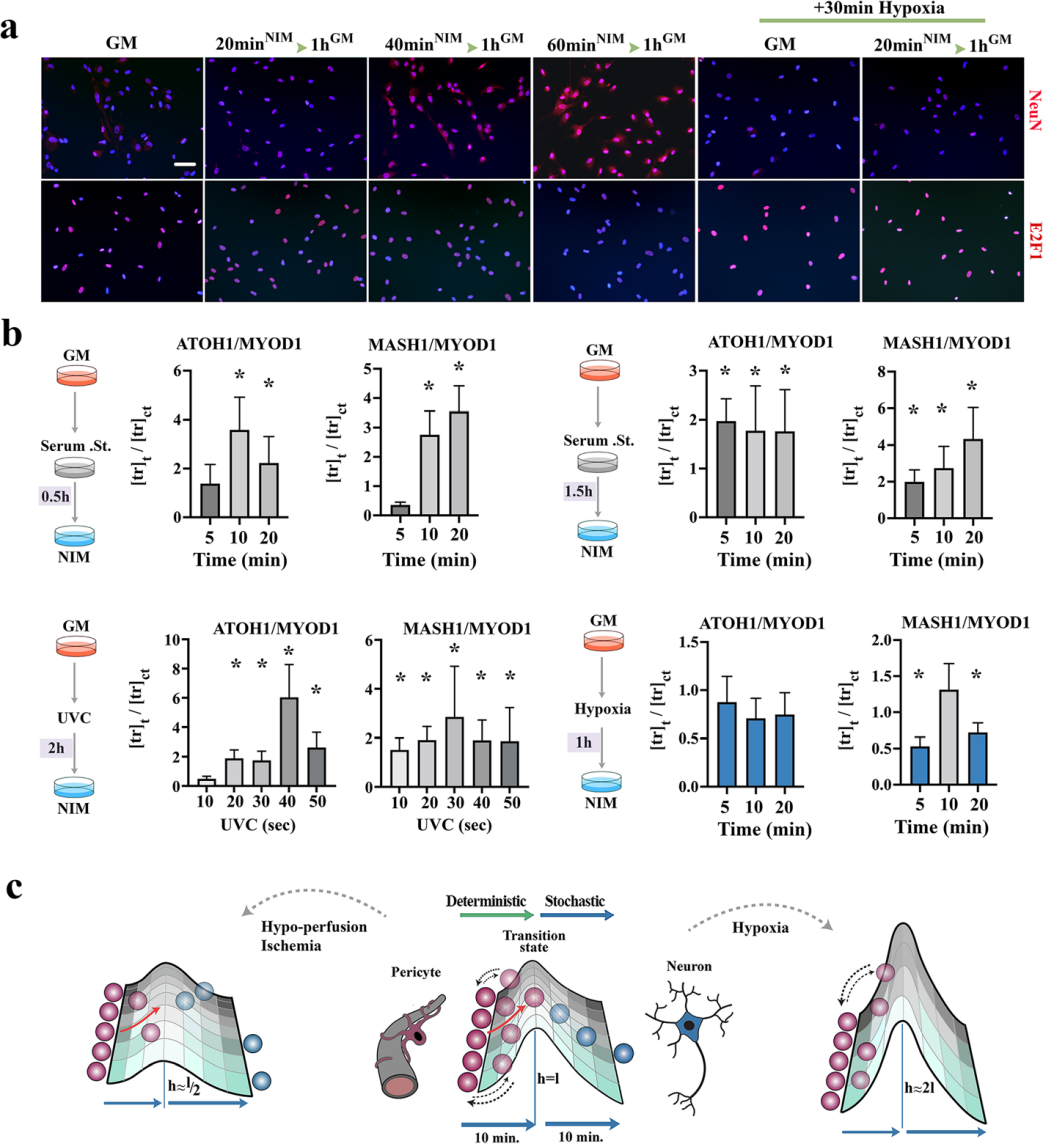
Transdifferentiation efficiency is modulated by exogenous and endogenous cues. **a** Immunohistochemical identification of NeuN^+^ transdifferentiated cells in TD-chase experiments wherein pericytes were exposed to neural induction medium (NIM) for 20, 40, and 60 min and then returned to growth medium for 16 h. In TD-chase experiment, NueN^high^/E2F1^low^ profile suggested cell cycle exit and completion of neural lineage reprogramming. Right columns demonstrate the outcome of TD-chase in pericytes conditioned by exposure to hypoxia (*t* = 30 min) prior to TD. **b** qPCR quantification of the ratio of ATOH-1 and MASH-1 to MYOD1 as a proxy for TD tendency in pericytes conditioned via serum starvation, hypoxia, and UVC irradiation prior to TD, as per schematic images. *X*-axis shows time points after the induction of TD. * indicates *p* < 0.01. **c** Schematic images summarize the role of exogenous and endogenous cues in programming the transdifferentiation tendency of pericytes. Height of the TD landscape is an approximation of TD propensity of pericytes

Finally, we attempted to estimate the transdifferentiation tendency (i.e., likelihood of TD or *P*^TD^) of individual pericytes by combined application of experimentation and mathematical modelling. In experiments termed TD-chase, pericytes were cultured in neural induction medium (NIM) for 20, 40, and 60 min and then were re-incubated in growth medium for *t* = 16 h in order to restrict TD to the studied temporal windows (Fig. 7a). In a TD-chase experiment, conversion from NueN^low^/E2F1^high^ state to NueN^high^/E2F1^low^ state was considered to indicate cell cycle exit and a commitment to neural differentiation. TD-chase experiments disclosed that two non-overlapping 20-min cycles were sufficient to enrich the transdifferentiated NueN^high^/E2F1^low^ cells to > 90% of the original pericytic population (Fig. 7a). This was also consistent with transcriptional profiling of pro-neural TFs fingerprinted at 5-min intervals (Fig. 1c), indicating that maximum expression of ATOH-1 and MASH-1 is achieved at *t* = 40 min after induction of transdifferentiation. Therefore, transdifferentiation rate was conservatively estimated as 50% of population/20-min cycle. In order to convert the population-level TD rate to transdifferentiation likelihood (i.e., *P*^TD^) of individual pericytes, we modelled TD as a Poisson process with two key assumptions; that TD is a binary event and that TD of individual cells are independent from each other. Hence, the likelihood of resistance to TD or *P*^TD^ (*k* = 0) in Poisson probability distribution corresponds to:

$$ P(k)=\frac{m^k\times {e}^{-m}}{k!} $$

$$ P\left(k=0\right)={e}^{-m} $$

Where *m* is derived from the population-level rate of TD (*m* = 50% or 0.5). It then follows that

$$ P\left(k=0\right)={e}^{\left(-0.5\right)}=0.6 $$

And hence the propensity for TD of individual pericytes is estimated as *P*^TD^ ≈ 40%. Given that *P*^TD^ reflects stochasticity of R-loop induction, a *P*^TD^ ≈ 40% can also be interpreted as the likelihood of occurrence of R-loops in a TD-promoting manner. It is reassuring that ≈46% of R-loop^+^ loci in DRIP-seq are specific to transdifferentiating pericytes (Fig. 4e), closely approximating the estimated *P*^TD^ of individual pericytes based on Poisson distribution.

Armed with knowledge that hypoxia and serum starvation alter the baseline expression of pro-neural transcription factors in growth medium (Fig. 1a, b), we questioned if preconditioning the pericytes by exogenous cues would alter *P*^TD^ of these cells. Preconditioning the pericytes and repeating the TD-chase experiments revealed that while hypoxia alone reduces the TD propensity of pericytes, serum starvation sensitizes these cells to TD (Fig. 7b). We also reasoned that accumulation of genomic damage, and the resultant genomic instability would tip the balance of TD outcome in favor of neural TD. This is because pre-TD genomic damage preferentially affects pro-myogenic loci, while sparing the repressed pro-neurogenic loci. As anticipated, application of short pulses of UV irradiation prior to transdifferentiation (see “Methods”) led to enhanced TD propensity (Fig. 7b). It was concluded that exogenous and endogenous cues could alter the population-level TD propensity of pericytes (measured as ratio of pro-neural to pro-myogenic transcription factors) by an average of ≈2-fold as referenced to the transdifferentiation tendency of unconditioned pericytes (Fig. 7c). Notably, exposure to exogenous cues and stressors has the potential to program sublineage differentiation outcome subsequent to TD. Commitment to glial differentiation, for example, requires input from Notch-1 signalling pathway [66]. This pathway, on the other hand, not only is regulated by the history of exposure to stressors but it also records and carries the memory of exposure to subsequent cellular generations in pericytes [67]. While it remains to be proven, we propose that the programmability of neural TD by stressors provides an effective path for accessing the archived cellular memory of exposure to stressors and the guided regeneration of damaged neural tissues based on such archived memory.

## Discussion

In this study, we investigated the mechanistic basis for the invariant neural transdifferentiation of mural pericytes [14]. Findings supported the contention that pericytes remain poised for neural transdifferentiation in a metastable state in perfused microvasculature. That is, global transcriptional landscape not only shapes the functional identity of pericytes, but also stabilizes the functional memory by silencing the competing neural program in a repressed chromatin state. Global disruption of the transcriptional landscape of pericytes leads to rapid relaxation of higher-order chromatin topology, emergence of a competing neural transcriptome and initiation of transdifferentiation. The cascade of lineage reprogramming is completed by induction of R-loops and elimination of the smooth muscle transcriptome.

We demonstrate that the original identity (i.e., transcriptome) of pericytes is concealed and protected in a repressed higher-order chromatin state by transcriptional dynamics that generate the transient functional identity of these cells as contractile elements of the microvasculature. The strategy of restricting access to genetic information by regulating higher-order chromatin topology is commonly utilized in the prokaryotic world as a critical survival strategy [68]. In *Escherichia coli*, for example, negative supercoiling represses transcription from certain genomic loci (7% of the genome) whose products are only required in adaptation to unfavorable environmental conditions [35]. Such unfavorable environmental cues directly alter the transcriptional landscape of *E. coli* leading to relaxation of supercoiled chromatin and rapid expression of the suppressed genomic loci. The principle idea behind this concept stems from the observation that chromatin topology and transcriptional landscape affect each other in a reciprocal manner [69]. While higher-order chromatin configuration limits access to encoded genetic information, transcriptional dynamics stabilize open and repressed topological domains of the genome [69]. Our findings suggest that mutually exclusive chromatin topologies encode the pericytic and neuronal functional identities and interconversion of these topological states underpins neural transdifferentiation of pericytes. Due to the functional redundancy of pro-neurogenic and pro-myogenic transcription factors [70] and homology of the binding sites [71], lineage specification can only be achieved by encoding of the binding sites and the associated differentiation programs in mutually exclusive higher-order chromatin states [71]. In Phase-I, transdifferentiating pericytes, localized relaxation of the repressed chromatin state leads to a window of bistability where myogenic and neurogenic programs co-exist. In Phase II of TD, R-loop-mediated DNA cleavage leads to depletion of the myogenic transcriptome and emergence of monostability characterized by dominance of the neural transcriptome. The spatial distribution and temporal synchronicity of R-loops and hence the efficiency of TD, is programmed by the transcriptional landscape of transdifferentiating pericytes. While it remains to be proven, it seems plausible to postulate that integration of endogenous local cues could tailor the outcome of TD to accommodate the specific functional demands of a regenerating tissue. The programmability of Phase II of transdifferentiation could potentially be utilized in regenerative medicine to achieve the desired outcome.

## Conclusion

Our findings strengthen the demonstrated interplay between angiogenesis and neurogenesis [14, 22, 72] by providing a mechanistic basis for neural transdifferentiation of pericytes. We conclude that the transcriptional landscape of pericytes not only shapes the functional identity of this cell type in stable microvessels, but it also silences and protects the competing neural differentiation program by induction of a repressed metastable higher-order chromatin state. Destabilization of metastable higher-order chromatin topology of pericytes is sufficient for rapid emergence of the repressed neurogenic program. Completion of lineage reprogramming requires R-loop-mediated induction of DNA cleavage that remodels the smooth muscle transcriptional profile of pericytes prior to emergence of the neural transcriptome.

## Methods

### Reagents

All chemicals were purchased from Sigma-Aldrich Inc. unless stated otherwise. All primers were purchased from IDT DNA. The cdk-9 inhibitor, DRB (5,6-Dichlorobenzimidazole 1-β-D-ribofuranoside, Sigma), was applied at a final concentration of 40 μM [73]. The plasmids pEGFP-RNASEH1, pEGFP-RNASEH2A, pEGFP-RNASEH2B, and pEGFP-RNASEH2C were a gift from Andrew Jackson & Martin Reijns (RRIDs:Addgene_108699, Addgene_108700, Addgene_108697, Addgene_108698) [74]. N-cadherin in pCCL-c-MNDU3c-PGK-EGFP was a gift from Nora Heisterkamp (RRID:Addgene_38153) [75].

### Cell culture

Human neural pericytes were purchased from ScienCell (Carlsbad, CA; #1200). We have authenticated the primary brain pericytes using a combination of immunohistochemical fingerprinting and gene expression profiling [14, 76]. The cells are characterized by expression of cytoplasmic α-smooth muscle actin, secretory VEGFA, and nuclear MyoD. Human neural pericytes were cultured in Dulbecco’s modified Eagle’s medium/F12 (DMEM/F12) supplemented with 10% fetal calf serum, recombinant human FGF-2 20 ng/ml (R&D Systems, 233-FB), and Antibiotic-Antimycotic (100×, Life Technologies). The cells were cultured in T25 flasks and media changed on a daily basis. Pericytes were passaged every 24 h at 70–80% confluence stage. Hypoxia experiments were performed in an anaerobic chamber based on the timeframe provided in the main text. Serum starvation experiments were performed by elimination of FBS from the growth medium. In order to simulate the combined effect of absence of mechanical cues and enhanced reductive stress on induction of pro-neural transcription factors, we cultured pericytes in serum-free neurobasal medium (Gibco) containing the reductive N2 supplement (0.25 mM transferrin-bound Fe3+, redox potential of Fe3+/Fe2+: + 0.77 volts, Gibco) and GlutaMAX (Gibco).

### Gene expression analysis

RNA was isolated using RNeasy Mini Kit (Qiagen). After DNase treatment, reverse transcription of the extracted RNA was carried out using a mixture of 1 μL of oligo-dT, 4 μL of total RNA, 1 μL of dNTP Mix (10 mM each), 4 μL of 5× First-Strand synthesis Buffer, 1 μL of 0.1 M DTT, 1 μL of RNaseOUT (40 units/μL), and 1 μL of SuperScript-III reverse transcriptase (200 units). Reverse transcription was performed at 50 °C for 50 min followed by 55 °C for 15 min. RNA was subsequently digested with RNAase H. To design primers, gene sequence data and exon/intron boundaries were obtained from GenBank database (see Additional file 1: Table S3). In each of the primer sets, the common 3′ or 5′ primer spanned the adjacent exons to prevent amplification of genomic DNA. Real-time PCR (38 cycles) was performed using SensiFAST™ SYBR® Lo-ROX reagents (BIOLINE®). Reaction mix comprised of 2 μl of cDNA, 400 nM primers (1.5 μl/primer), 10 μl of 2× SensiFAST SYBR Lo-ROX Mix, and 5 μl of PCR-grade water on a Stratagene® Mx3000P real-time PCR instrument. The relative expression ratio of gene of interest (test:control) was then calculated using the efficiency (Eff.) values based on the method proposed by Pffafi as follows:

$$ Ratio=\frac{{\left({Eff}_{tar}\right)}^{\Delta {ct}_{tar}\left( control\hbox{-} test\right)}}{{\left({Eff}_{ref}\right)}^{\Delta {ct}_{ref}\left( control\hbox{-} test\right)}} $$

### UVC irradiation

Pericytes were transferred into a 6-well-plate and cultured for 1 day. A 30-watt UVC generator at a distance of 40 cm from the plate was used as a source of ionizing radiation. The UVC lamp was turned on for 5 × 10 s with 10-s intervals. The cells were then immediately supplemented with fresh growth medium.

### Programming of chromatin higher-order topology

To stabilize the higher-order topology of chromatin, pericytes were pre-incubated with 4,5′,8-trimethylpsoralen (TMP, Sigma, final concentration: 20 μg/mL) in growth medium for 10 min in order to allow intercalation of TMP into negatively supercoiled (underwound) chromatin domains [77, 78]. Cells were then placed on an ice bed and exposed to ≈3 kJ/m^2^ of 365 nm light for 30 s (ultra-high intensity UV-A lamp, Maxima: model ML-3500S) held at a distance of 20 cm measured from the surface of the light filter to the surface of culture dishes. TMP-containing medium was the replaced with fresh growth medium after 10 min and experiments were carried out as per text.

### Quantification of locus topology

To study the higher-order topology of loci of interest, pericytes undergoing transdifferentiation in induction medium were lysed at 2-min intervals. Subsequent to cell lysis, 4,5′,8-trimethylpsoralen (TMP, Sigma) was employed to intercalate into negatively supercoiled (underwound) DNA [77, 78]. The dishes containing the lysate were then placed on an ice bed and exposed to ≈3 kJ/m^2^ of 365 nm light for 40 s (ultra-high intensity UV-A lamp, Maxima: model ML-3500S) held at a distance of 15 cm measured from the surface of the light filter to the surface of cell lysate-containing dish. The extraction of DNA was then completed using a DNA isolation kit (ISOLATE II Genomic DNA Kit, meridian Biosciences) according to the manufacturer’s instructions. The isolated DNA was digested with MNase (2 U per million cells, NEB) for 5 min at room temperature. The enzyme was inactivated by adding EGTA. Final reaction products were analyzed by DNA gel electrophoresis (1%) in the presence of chloroquine (5 μg/ml, Sigma) to distinguish differences in negative supercoiling densities and in control native gels. Four genomic fractions corresponding to the zones I–IV of Fig. 2c (refer to the main text) were then cut and purified rom the gel using Wizard SV Gel and PCR Clean-Up System (Promega). Isolated fractions were then incubated in a denaturing solution (0.5 M NaOH, 1.5 M NaCl) at 65 °C for 60 min to reverse psoralen crosslink. Specific PCR primers (Additional file 1: Table S4) were designed to quantify the distribution of the loci of interest (LoI) in four isolated genomic fractions. Real-time PCR (38 cycles) was performed using SensiFAST™ SYBR® Lo-ROX reagents (BIOLINE®). Reaction mix comprised of 2 μl of gDNA, 400 nM primers, 10 μl of 2× SensiFAST SYBR Lo-ROX Mix, and 5 μl of PCR-grade water on a Stratagene® Mx3000P real-time PCR instrument. Normalized representations of topological states of the LoI (Fig. 2d of the main text) in zones I–IV (*Z*_*i*_, *i*: 1–4) were generated based on the following formula:

$$ \boldsymbol{LoI}\_{\boldsymbol{Z}}_{\boldsymbol{i}}=\frac{{\mathbf{2}}^{\boldsymbol{\Delta  Ct}\left({\boldsymbol{Z}}_{\boldsymbol{i}}-{\boldsymbol{Z}}_{\mathbf{1}}\right)}}{\sum \limits_{\boldsymbol{i}=\mathbf{1}}^{\mathbf{4}}{\mathbf{2}}^{\boldsymbol{\Delta  Ct}\left({\boldsymbol{Z}}_{\boldsymbol{i}}-{\boldsymbol{Z}}_{\mathbf{1}}\right)}} $$

where LoI_*Z*_*i*_ (*i*: 1–4) represent the normalized distribution of a locus of interest in zones I–IV corresponding to various higher-order topological states that form a gradient from sc^−^-dominated zone I, with low electrophoretic mobility, to sc^+^-dominated zone IV, with high electrophoretic mobility.

### RNA-Seq analysis

RNA sequencing was performed by Ramaciotti Centre for Genomics (UNSW Sydney, Australia). Total RNA was extracted using RNeasy Mini Kit (Qiagen). RNA integrity was evaluated using the Agilent 2100 Bioanalyzer (Agilent). After enrichment of Poly-A^+^ mRNA, the libraries were constructed using TruSeq Stranded mRNA Library Prep Kit (Illumina). Then the libraries were sequenced on HiSeqTM 2500 sequencing platform (Illumina). To perform transcript-level expression analysis, RNA-seq reads were mapped to *H. sapiens* GRCh38 reference transcriptome using Hisat2. StringTie was utilized to assemble and quantify expressed transcripts. The transcript abundance and differential expression tables were generated using Ballgown. Finally, the statistical analysis of between-group differences was performed using DESeq R package. Gene ontology (GO) enrichment analysis was done using the Metascape, NetworkAnalyst, and the Reactome databases. After analysis, GO clusters with *p* < 0.01 were selected and the log_2_ (fold_change) for transcripts in the cluster were extracted from the Edge R table and graphed as a scatter plot where the values are normalized to the control group.

### ChIP-seq and DRIP-seq

ChIP-seq was performed as described elsewhere [79]. For ChIP-seq and DRIP-seq (DNA-RNA immunoprecipitation), 5 × 10^6^ cells were fixed with ice-cold 1% formaldehyde for 10 min at RT and crosslinking was quenched with 125 mM glycine for 5 min at RT. Sonication was performed using a Covaris S220 to create ≈200–300 bp fragments. For ChIP-seq and DRIP-seq, chromatin was then immunoprecipitated with anti-γH2AX (Abcam) and S9.6 antibodies, respectively. Chromatin was then incubated overnight at 4 °C with protein G-coated Sepharose beads (Sigma). Beads were then washed twice with each of the following buffers: low-salt, high-salt, and TE wash buffers. DNA was subsequently eluted and digested with proteinase K (20 μg/μl) for 2 h at 55 °C and incubated overnight at 65 °C to reverse crosslinks. Pure DNA was isolated using the ISOLATE II Genomic DNA Kit and 15–30 ng of size selected DNA fragments were used to produce ChIP-seq libraries (Illumina TruSeq ChIP Library Preparation Kit). ChIP-seq libraries were processed through a high-throughput sequencing pipeline using Illumina platform. Reads were mapped to the human genome (*H. sapiens* GRCh38) using Hisat2. The MACS software [80] was used to identify the peaks of sequence reads.

### ATAC-seq

In total, 50,000 nuclei from control and transdifferentiating pericytes were used for ATAC-seq as described elsewhere [81] with a slight modification. Due to rapid pace of TD and a requirement for sampling at short intervals (2 min), cell lysis and crude nuclei preparation were done on culture plates and without harvesting the adherent cells. Paired-end ATAC-seq libraries were sequenced by the Australian Genome Research Facility and based on Illumina NovaSeq platform.

### Immunoblotting

Extracted proteins were separated by PAGE using gradient 5 to 12% minigels, transferred to 0.2-μm nitrocellulose membranes (Bio-Rad) and blocked for ≥ 2 h with 3% bovine serum albumin (Sigma) in 0.1 M Tris buffered salts solution pH 7.4 (TBS). Blotted antigens were incubated with primary antibodies in 0.05% Tween20/TBS for 2 h, washed and subsequently incubated with HRP-conjugated secondary antibody (goat-anti rabbit/mouse IgG, DAKO, Denmark) diluted 1:1500 in Tween20/TBS for 2 h. Bound antibody was visualized using SuperSignal West Pico PLUS Chemiluminescent Substrate (Thermo Scientific).

### Immunohistochemistry

Cells were processed as described previously [14, 82, 83] and fixed in 2% paraformaldehyde/5% sucrose in 0.02 M phosphate buffer pH:7.4 (680 mOsm) for 4 h at 4 °C. After blocking in incubation buffer containing 0.1 M PBS, 1% BSA, 0.1% Tween-20, and 5% normal goat serum (for detection with rabbit Abs) or 5% normal rabbit serum (for detection with mouse Abs) for 40 min, sections were incubated with the primary antibodies overnight at 4 °C and secondary antibodies for 1 h at room temperature. Specificity controls were carried out by incubating sections with rabbit or mouse IgG negative control antibodies.

### Electron microscopy

For TEM analysis, samples were fixed in Karnovsky’s fixative overnight at room temperature followed by post-fixation in OsO4 for 1 h. Preparations were dehydrated in graded alcohols and embedded in low viscosity resin (TAAB Laboratory and Microscopy, UK). Ultrathin sections were mounted on Pioloform/formvar-coated slot grids, stained in uranyl acetate and lead citrate, and examined in a Phillips CM120 BioTWIN electron microscope.

### Confocal and multiphoton imaging

Fluorescence imaging of samples was performed using an Olympus FV1000 confocal laser scanning microscope with ✕ 40 water-immersion and ✕ 60 oil-immersion objectives (Carl Zeiss; Jena, Germany). High-magnification images were recorded with a 0.45 μm *z*-step and 0.125 μm *x*/*y* pixel size. Fluorescence imaging of 300-μm sections was achieved using a Leica SP5 II multiphoton microscope.

### RNA interference

For small interfering RNA (siRNA)-mediated knockdown of CDK9, cells were electroporated with a cocktail containing 200 nM of two siRNAs. The sequences of siRNAs targeting nAS25 are as follows:

- CDK9-siRNA 1:
  Sense: 5′-rGrGrGrArCrUrUrGrArUrUrGrUrCrArArGrUrCrArCrUrGGA-3′
  Antisense: 5′-rUrCrCrArGrUrGrArCrUrUrGrArCrArArUrCrArArGrUrCrCrCrArA-3′
- CDK9-siRNA 2:
  Sense: 5′-rCrUrUrCrArGrCrUrUrCrUrArArArArCrArCrGrArGrArATG-3′
  Antisense: 5′-rCrArUrUrCrUrCrGrUrGrUrUrUrUrArGrArArGrCrUrGrArArGrGrA-3′

For electroporation in RNAi, cells were harvested, mixed with Dsi-RNAs (i.e., siRNAs) and resuspended in 400 μL of electroporation buffer (10^6^ cells/400 μL). Electroporation buffer comprised 20 mM HEPES, 135 mM KCl, 2 mM MgCl2, 0.5% Ficoll 400, and 2 mM ATP/5 mM glutathione (pH 7.6). Electroporation was carried out at 1700 V/cm, 700 μs, four pulses at 1-s intervals.

### Crispr-Cas9 global genome editing with TDi-RNA

To design the TDi-RNA, a genic cluster (*n* = 469 genes) was identified (Fig. 6a, Additional file 1: Table S2) based on the principle that conserved homologous regions longer than 13 bp are unlikely to be randomly seeded into the genome [65]. Gene ontology analysis revealed that members of the identified cluster were involved in regulation of focal adhesion and stabilization of actomyosin (Fig. 6b). An sgRNA was designed with complementarity (> 13 bp) to the seed regions distributed non-randomly in the identified genic cluster. The sgRNA sequence was mA*mC*mU*GUGAGUGUGGAGACCUUGUUUUAGAGCUAGAAAUAGCAAGUUAAAAUAAGGCUAGUCCGUUAUCAACUUGAAAAAGAGGCACCGGUCGGUGCmU*mU*mU*rU. The sgRNA (termed TDi-RNA based on the novel design principle and function), Alt-R® S.p. HiFi Cas9 Nuclease V3 and Alt-R® S.p. Cas9 D10A Nickase V3 were purchased from IDTDNA. SgRNA and the enzymes were resuspended in Nuclease-Free Duplex Buffer and Cas9 buffer (20 mM HEPES; 150 mM KCI, pH 7.5), respectively. Ribonucleoprotein (RNP) complex was prepared by combining the sgRNA (final concentration: 120 pmol) and Cas9 enzyme (final concentration: 104 pmol) and incubation at RT for 20 min. For electroporation, pericytes in growth medium were harvested, mixed with RNP, and resuspended in 400 μL of electroporation buffer (10^6^ cells/400 μL). Electroporation buffer comprised 20 mM HEPES, 135 mM KCl, 2 mM MgCl2, 0.5% Ficoll 400, and 2 mM ATP/5 mM glutathione (pH 7.6). Naked pre-miRNA-4673 was applied at a final concentration of 200 nM. Electroporation was carried out at 1700 V/cm, 700 μs, four pulses at 1-s intervals. After introduction of the TDi-RNA (sgRNA) and Cas9 nuclease or nickase, pericytes were returned to growth medium and incubated for an additional 12 h.

### Statistics and reproducibility

SPSS statistical software (SPSS v.16, Chicago, Illinois, US) was used for the statistical analysis of data. The relative expression levels of genes of interest were compared using univariate ANOVA and non-parametric Mann-Whitney *U* test. In the present study, a *p* value < 0.01 was considered as statistically significant.

## Supplementary Information

**Additional file 1 **: **Figure S1**. Immunostaining of stable long-term cultures of transdifferentiated pericytes. **Figure S2**. Ki-67 immunoreactivity of transdifferentiating pericytes. **Figure S3**. Transcriptional fingerprinting of genes of interest in transdifferentiating pericytes. **Figure S4**. IHC profile of transdifferentiating pericytes. **Table S1**. PCR primers utilised in fingerprinting VEGFA locus. **Table S2**. Chromosomal distribution of genomic loci with sequence homology to sgRNA (TDi-RNA). **Table S3**. Transcript-specific primers used in qPCR. **Table S4**. Specific primers used in quantification of locus topology.

**Additional file 2.** Peer review history.

## Data Availability

All data generated or analyzed during this study are included in this published article and its supplementary information files. The RNA-seq, ChIP-seq, DRIP-seq, and ATAC-seq data described in this study are deposited to the Gene Expression Omnibus (GEO) repository (GSE187020) [84]. The accession number for the RNA-seq data reported in this paper is GEO: GSE187019. The accession number for the ATAC-seq data is GEO: GSE187016. The accession numbers for the ChIP- and DRIP-seq data reported in this paper are GEO: GSE187017 and GSE187018.
