# Supplementary material for: Programmed genomic instability regulates neural transdifferentiation of human brain microvascular pericytes
Source: Genome Biol. 2021 Dec 9;22:334. doi: 10.1186/s13059-021-02555-0 (PMC8656028; doi:10.1186/s13059-021-02555-0)
Supplement: Supplementary file 1 — Additional file 1 : Figure S1. Immunostaining of stable long-term cultures of transdifferentiated pericytes. Figure S2. Ki-67 immunoreactivity of transdifferentiating pericytes. Figure S3. Transcriptional fingerprinting of genes of interest in transdifferentiating pericytes. Figure S4. IHC profile of transdifferentiating pericytes. Table S1. PCR primers utilised in fingerprinting VEGFA locus. Table S2. Chromosomal distribution of genomic loci with sequence homology to sgRNA (TDi-RNA). Table S3. Transcript-specific primers used in qPCR. Table S4. Specific primers used in quantification of locus topology. [file 13059_2021_2555_MOESM1_ESM.pdf]

Additional file 1

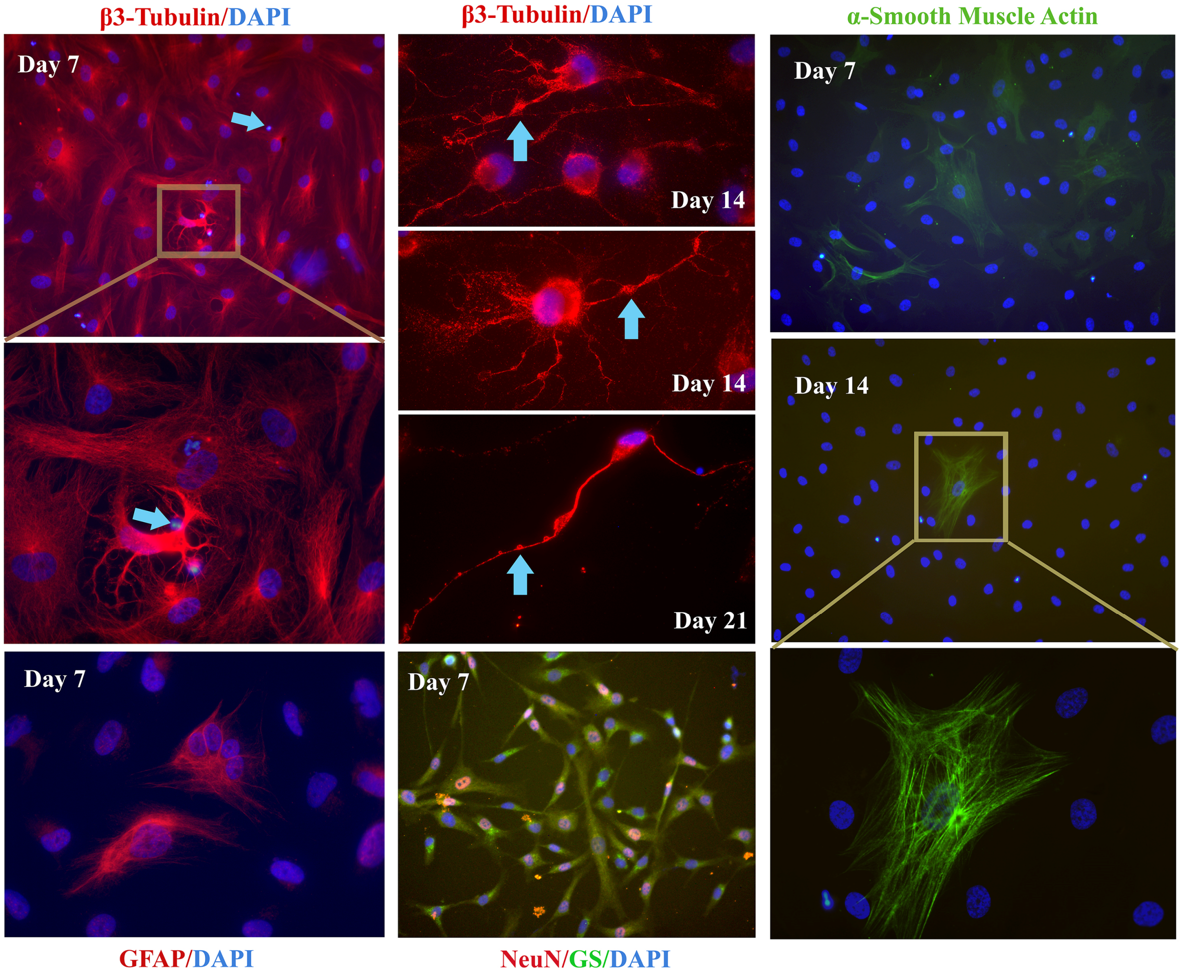

Fig. S1 | Immunostaining of stable long-term cultures of transdifferentiated pericytes. The left column shows near-complete transdifferentiation of pericytes into ß3-tubulin^{+} neurons after 7 days in culture (arrows: extranuclear DNA), concurrent with the depletion of smooth muscle actin^{+} pericytes (right column). The lack of apoptotic nuclei and cell death is a characteristic feature of neural TD of pericytes. The middle column shows gradual appearance of dendritic spines (arrows, a feature of mature functional neurons) from day 14 to day 21 post-TD. Bottom IHC pannels show GFAP^{+}/Glutamine Synthetase^{+} (GS) glial cells that are generated from transdifferentiated pericytes.

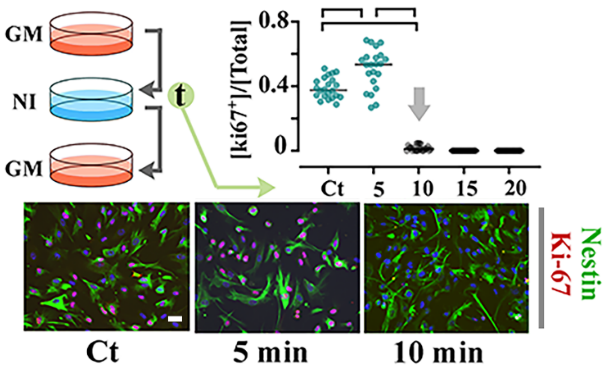

Fig. S2 | Ki-67 immunoreactivity of transdifferentiating pericytes. Scatter graph (bottom) shows Ki-67^{+} cycling pericytes after transient incubation in neural induction medium for t=5-20 min and re-incubation in growth medium for 18h (brackets: $p<0.01$). Note that after 10 min of incubation in NI, pericytes become non-response to mitogenic cues as evidevced by complete absence of Ki67 immunoreactivity, even after 18 hours of incubation in growth medium.

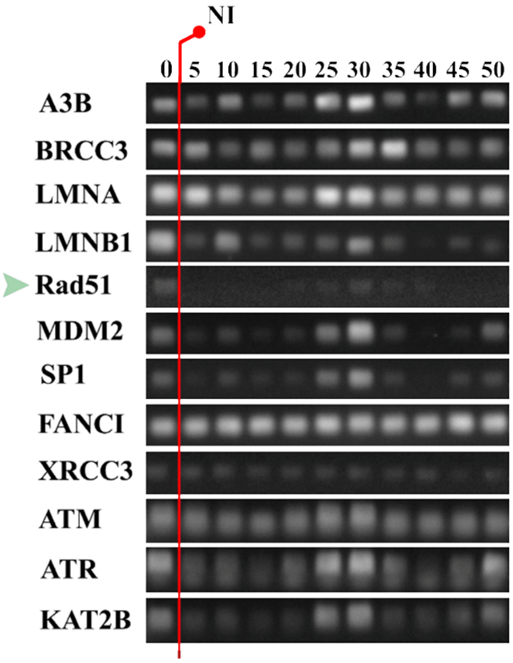

Fig. S3 | Agarose gels show transcriptional fingerprinting of genes of interest in transdifferentiating pericytes (t=0-50 min) (https://doi.org/10.6084/m9.figshare.16912024). Note that Rad51 is downregulated immediately after initiation of transdifferentiation.

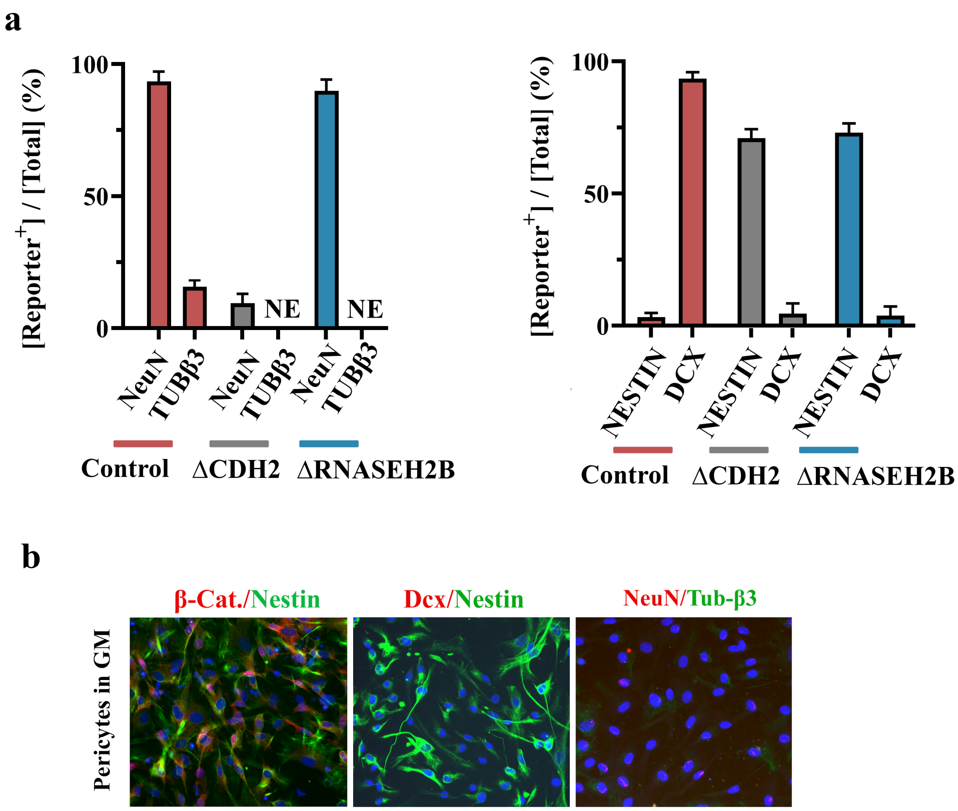

Fig. S4 | IHC profile of transdifferentiating pericytes. a, Quantification of IHC immunoreactivity of selected neuronal differentiation markers subsequent to forced expression of CDH2 and RNASEH2B. **b,** Expression of NeuN, Dcx, Nestin and ß3-Tubulin in control pericytes prior to transdifferentiation in growth medium.

Table S1. PCR primers utilised in fingerprinting VEGFA locus.

Primer set	Oligos	Primer sequence
**VEGFA1**	F-primer	GGTAGCTCGGAGGTCGTGG
	R-primer	AGCCTCTGGCTTCTCCAAGTCTT
**VEGFA2**	F-primer	ATGGGGTTCCCTGTCCTCTCA
	R-primer	GGTCTCGATTGGATGGCAGTAGC
**VEGFA3**	F-primer	CCATCCTGTGTGCCCCTGAT
	R-primer	GCTTTCTCCGCTCTGAGCAAGG
**VEGFA4**	F-primer	CAAGGCGAGGCAGCTTGAGT
	R-primer	GAGTGTCCCATCAGTGGTGACC

Table S2. Chromosomal distribution of genomic loci with sequence homology to sgRNA (TDi-RNA). The length of seed regions and presence or absence of flanking canonical PAM sequence are shown in the last two columns.

Chromosome	start	end	Gene	homology	PAM
Chr6	43780410	43780432	VEGFA	23	1
Chr6	69904993	69905007	COL19A1	15	0
Chr6	39059077	39059090	GLP1R	14	0
Chr6	4781585	4781597	CDYL	13	0
Chr6	11249909	11249921	NEDD9	13	0
Chr6	25692515	25692527	SCGN	13	0
Chr6	28527041	28527053	GPX5	13	0
Chr6	34124960	34124976	GRM4	16	1
Chr6	39861985	39861997	DAAM2	13	0
Chr6	55870366	55870382	BMP5	16	0
Chr6	97059277	97059289	KLHL32	13	0
Chr6	108327975	108327987	AFG1L	13	1
Chr6	130048433	130048445	L3MBTL3	13	0
Chr6	133283853	133283865	EYA4	13	1
Chr6	144302191	144302207	UTRN	16	0
Chr6	146126085	146126097	GRM1	13	0
Chr6	161661297	161661309	PRKN	13	0
Chr6	165813541	165813553	PDE10A19	13	1
Chr7	108080392	108080406	LAMB4	15	0
Chr7	144403642	144403656	NOBOX	15	0
Chr7	151062136	151062150	SLC4A2	15	0
Chr7	29032130	29032143	CPVL	14	1
Chr7	29259396	29259409	CHN2	14	0
Chr7	48366754	48366767	ABCA13	14	0
Chr7	131262891	131262904	MKLN1	14	0
Chr7	157188963	157188976	UBE3C	14	0
Chr7	158001343	158001356	PTPRN2	14	0
Chr7	8215230	8215242	ICA1	13	0
Chr7	28668467	28668479	CREB5	13	1
Chr7	31656803	31656815	ITPRID1	13	1

Chr7	37714162	37714174	GPR141	13	0
Chr7	38257191	38257203	TARP	13	0
Chr7	38803716	38803728	VPS41	13	0
Chr7	45625771	45625783	ADCY1	13	1
Chr7	87054925	87054937	KIAA1324L	13	0
Chr7	91950653	91950665	AKAP9	13	0
Chr7	112868766	112868778	BMT2	13	0
Chr7	139714153	139714165	HIPK2	13	0
Chr11	60335123	60335138	MS4A6E	16	0
Chr11	20857550	20857564	NELL1	15	0
Chr11	60181498	60181484	MS4A6A	15	0
Chr11	14034391	14034408	SPON1	17	1
Chr11	70699693	70699706	SHANK2	14	0
Chr11	71071252	71071265	SHANK2	14	0
Chr11	76004114	76004127	UVRAG	14	0
Chr11	84270702	84270715	DLG2	14	0
Chr11	2390765	2390777	CD81	13	0
Chr11	7064610	7064622	NLRP14	13	1
Chr11	11994135	11994147	DKK3	13	0
Chr11	17099317	17099329	PIK3C2G	13	1
Chr11	20029112	20029124	NAV2	13	0
Chr11	21204775	21204787	NELL1	13	1
Chr11	27351250	27351262	CCDC34	13	0
Chr11	34305205	34305217	ABTB2	13	0
Chr11	46368441	46368453	DGKZ	13	0
Chr11	61278044	61278056	VWCE	13	0
Chr11	62132643	62132655	INCENP	13	1
Chr11	70135304	70135320	ANO1	16	1
Chr11	73383820	73383832	RELT	13	0
Chr11	78705207	78705219	TENM4	13	0
Chr11	99852873	99852885	CNTN5	13	0
Chr11	100014902	100014914	CNTN5	13	0
Chr11	115205188	115205204	CADM1	16	0

Chr11	120847246	120847230	GRIK4	16	0
Chr11	124874907	124874919	ROBO3	13	1
Chr11	125641404	125641416	CHEK1	13	0
Chr11	125950287	125950299	VSIG10L	13	1
Chr11	128915389	128915401	KCNJ5	13	0
Chr11	133054728	133054740	OPCML	13	0
Chr4	143613401	143613417	FREM3	17	0
Chr4	150790040	150790055	LRBA	16	0
Chr4	112411942	112411956	ALPK1	15	1
Chr4	147747560	147747574	ARHGAP10	15	0
Chr4	1305226	1305239	MAEA	15	0
Chr4	7046694	7046707	TADA2B	14	0
Chr4	15703457	15703470	BST1	14	0
Chr4	42399630	42399643	SHISA3	14	0
Chr4	119179925	119179938	MYOZ2	14	1
Chr4	142076089	142076102	INPP4B	14	0
Chr4	150218603	150218620	DCLK2	17	0
Chr4	154490246	154490259	DCHS2	14	0
Chr4	5367997	5368009	STK32B	13	0
Chr4	7312622	7312634	SORCS2	13	0
Chr4	8027840	8027852	ABLIM2	13	1
Chr4	8608746	8608758	CPZ	13	0
Chr4	23877914	23877926	PPARGC1A	13	0
Chr4	38030454	38030466	TBC1D1	13	1
Chr4	41475090	41475102	LIMCH1	13	0
Chr4	48131727	48131743	TXK	16	0
Chr4	69933715	69933727	CSN1S1	13	0
Chr4	74219380	74219392	MTHFD2L	13	1
Chr4	86050505	86050517	MAPK10	13	0
Chr4	93221386	93221398	GRID2	13	0
Chr4	102324932	102324944	SLC39A8	13	0
Chr4	108992675	108992691	COL25A1	16	0
Chr4	109740757	109740769	CFI	13	0

Chr4	150536782	150536794	LRBA	13	0
Chr4	153621162	153621174	TMEM131L	13	0
Chr4	182717484	182717496	TENM3	13	0
Chr8	1358452	1358466	DLGAP2	15	0
Chr8	117919054	117919067	EXT1	14	0
Chr8	124561560	124561573	MTSS1	14	0
Chr8	1669708	1669720	DLGAP2	13	0
Chr8	3333649	3333637	CSMD1	13	0
Chr8	10077268	10077280	MSRA	13	0
Chr8	14282307	14282319	SGCZ	13	0
Chr8	17608784	17608796	PDGFRL	13	0
Chr8	18869158	18869170	PSD3	13	0
Chr8	22520315	22520327	PPP3CA	13	1
Chr8	22613608	22613620	CCAR2	13	0
Chr8	35728743	35728755	UNC5D	13	0
Chr8	38347643	38347655	NSD3	13	0
Chr8	99461297	99461309	VPS13B	13	0
Chr8	115453971	115453983	TRPS1	13	0
Chr8	129866048	129866060	FAM49B	13	0
Chr8	138269269	138269281	FAM135B	13	0
Chr8	140748037	140748049	PTK2	13	0
Chr8	144469539	144469551	KIFC2	13	0
Chr10	125929332	125929348	FANK1	17	0
Chr10	106625284	106625298	SORCS1	15	1
Chr10	17001171	17001184	CUBN	14	1
Chr10	17689297	17689310	STAM	14	0
Chr10	71462502	71462519	CDH23	17	0
Chr10	103836676	103836663	SH3PXD2A	14	0
Chr10	124606436	124606449	LHPP	14	0
Chr10	630075	630087	DIP2C	13	0
Chr10	5106332	5106344	AKR1C3	13	0
Chr10	17021692	17021708	CUBN	16	0
Chr10	24483338	24483354	KIAA1217	16	0

Chr10	32844487	32844503	CCDC7	16	0
Chr10	51133623	51133639	PRKG1	16	0
Chr10	73520311	73520323	USP54	13	0
Chr10	74382575	74382587	ADK	13	1
Chr10	75015142	75015154	KAT6B	13	0
Chr10	76323577	76323589	LRMDA	13	1
Chr10	79403411	79403423	ZCCHC24	13	0
Chr10	79710972	79710984	NUTM2B	13	0
Chr10	79849093	79849105	NUTM2E	13	0
Chr10	87233195	87233207	NUTM2A	13	0
Chr10	87365411	87365423	NUTM2D	13	0
Chr10	100919624	100919636	SLF2	13	0
Chr10	101828467	101828479	KCNIP2	13	0
Chr10	102517906	102517918	SUFU	13	0
Chr10	104041462	104041474	COL17A1	13	1
Chr10	116104026	116104038	GFRA1	13	0
Chr10	126667575	126667587	C10orf90	13	0
Chr10	129516897	129516909	MGMT	13	0
Chr10	131178597	131178609	TCERG1L	13	0
Chr10	131243177	131243189	TCERG1L	13	0
Chr2	143441670	143441685	ARHGAP15	16	0
Chr2	28536144	28536158	PLB1	15	1
Chr2	201406783	201406797	TRAK2	15	0
Chr2	48754879	48754892	LHCGR	14	0
Chr2	196414500	196414513	HECW2	14	0
Chr2	23126377	23126389	LOC107985792	13	1
Chr2	30782010	30782022	CAPN13	13	0
Chr2	33064478	33064490	LTBP1	13	0
Chr2	37301176	37301188	PRKD3	13	0
Chr2	38061011	38061027	RMDN2	16	1
Chr2	42342184	42342196	COX7A2L	13	0
Chr2	42669674	42669686	MTA3	13	0
Chr2	47056130	47056142	TTC7A	13	1

Chr2	48501000	48501016	PPP1R21	16	0
Chr2	50551279	50551291	NRXN1	13	0
Chr2	56233462	56233474	CCDC85A	13	1
Chr2	63236282	63236294	WDPCP	13	0
Chr2	73104691	73104703	RAB11FIP5	13	1
Chr2	75511462	75511474	EVA1A	13	0
Chr2	77099901	77099913	LRRTM4	13	0
Chr2	80263893	80263905	CTNNA2	13	0
Chr2	84740871	84740883	DNAH6	13	0
Chr2	98222016	98222028	VWA3B	13	0
Chr2	98553609	98553621	INPP4A	13	0
Chr2	99952816	99952828	AFF3	13	0
Chr2	100970411	100970423	NPAS2	13	0
Chr2	114987788	114987800	DPP10	13	0
Chr2	115506835	115506847	DPP10	13	0
Chr2	134459610	134459622	TMEM163	13	0
Chr2	137117837	137117849	THSD7B	13	1
Chr2	137498891	137498903	THSD7B	13	1
Chr2	138679132	138679144	NXPH2	13	0
Chr2	140922910	140922926	LRP1B	16	0
Chr2	148696061	148696073	EPC2	13	0
Chr2	172870410	172870422	RAPGEF4	13	0
Chr2	184911672	184911684	ZNF804A	13	0
Chr2	192049541	192049553	TMEFF1	13	0
Chr2	202911261	202911273	WDR12	13	0
Chr2	216643897	216643909	IGFBP2	13	0
Chr2	219375302	219375314	DNPEP	13	0
Chr2	221526491	221526503	EPHA4	13	0
Chr2	224989039	224989051	DOCK10	13	0
Chr2	228065099	228065111	SPHKAP	13	1
Chr2	232653758	232653770	EFHD1	13	0
Chr2	236248829	236248841	ASB18	13	0
Chr2	239159703	239159715	HDAC4	13	0

Chr2	241321948	241321960	SEPT2	13	0
Chr3	48585542	48585556	COL7A1	15	0
Chr3	154381009	154381023	GPR149	15	0
Chr3	4784924	4784937	ITPR1	14	0
Chr3	13193240	13193253	IQSEC3	14	0
Chr3	30823418	30823431	GADL1	14	1
Chr3	42699362	42699375	HHATL	14	0
Chr3	94036506	94036519	ARL13B	14	0
Chr3	142724894	142724907	TRPC4	14	0
Chr3	9726398	9726410	CPNE9	13	1
Chr3	14663076	14663088	CCDC174	13	0
Chr3	18391762	18391774	SATB1	13	0
Chr3	21684073	21684085	ZNF385D	13	1
Chr3	23547267	23547283	UBE2E2	16	0
Chr3	30823294	30823306	GADL1	13	0
Chr3	31801496	31801508	OSBPL10	13	1
Chr3	40129562	40129574	MYRIP	13	0
Chr3	47001560	47001572	NBEAL2	13	0
Chr3	48525314	48525326	PFKFB1	13	1
Chr3	51935581	51935593	RRP9	13	0
Chr3	54140865	54140877	CACNA2D3	13	0
Chr3	54619018	54619030	CACNA2D3	13	1
Chr3	68293760	68293772	FAM19A1	13	1
Chr3	72391376	72391388	RYBP	13	0
Chr3	100309434	100309446	TBC1D23	13	0
Chr3	100958304	100958316	ABI3BP	13	0
Chr3	108963864	108963876	MORC1	13	1
Chr3	114011911	114011923	CCDC191	13	0
Chr3	123394451	123394463	ADCY5	13	0
Chr3	123769476	123769488	MYLK	13	0
Chr3	123979278	123979294	ROPN1	16	0
Chr3	124272887	124272899	KALRN	13	0
Chr3	125973236	125973252	ROPN1B	16	0

Chr3	127933375	127933387	KBTBD12	13	1
Chr3	129104774	129104786	RAB43	13	0
Chr3	130619376	130619388	COL6A6	13	0
Chr3	133888210	133888222	RAB6B	13	1
Chr3	135058501	135058513	EPHB1	13	0
Chr3	169099324	169099340	MECOM	16	0
Chr3	175256601	175256613	NAALADL2	13	0
Chr3	194099212	194099224	LOC102724877	13	0
Chr5	111869190	111869203	NREP	14	0
Chr5	167579663	167579676	TENM2	14	0
Chr5	178512977	178512990	COL23A1	14	0
Chr5	1319518	1319530	CLPTM1	13	0
Chr5	16132086	16132102	MARCH11	16	0
Chr5	64228012	64228024	RNF180	13	0
Chr5	81481834	81481846	SSBP2	13	0
Chr5	84174114	84174130	EDIL3	16	0
Chr5	119333278	119333294	TNFAIP8	16	0
Chr5	147263369	147263381	STK32A	13	0
Chr5	150370101	150370113	TCOF1	13	0
Chr5	158950146	158950158	EBF1	13	1
Chr5	163517427	163517439	MAT2B	13	0
Chr5	168020761	168020773	TENM2	13	0
Chr5	170628352	170628364	KCNIP1	13	1
Chr5	172389389	172389401	SH3PXD2B	13	0
Chr5	172663602	172663614	NEURL1B	13	0
Chr5	176413177	176413189	ARL10	13	0
Chr5	176968952	176968964	UIMC1	13	1
Chr5	179122220	179122232	ADAMTS1	13	0
Chr5	179832289	179832309	SQSTM1	19	0
Chr9	116912243	116912258	ASTN2	16	0
Chr9	15504910	15504923	PSIP1	14	0
Chr9	98392506	98392519	GABBR2	14	0
Chr9	114077491	114077504	AMBP	14	0

Chr9	132483515	132483532	CFAP77	17	0
Chr9	133960369	133960382	VAV2	14	0
Chr9	137272080	137272093	NELFB	14	1
Chr9	26880160	26880172	CAAP1	13	0
Chr9	34568721	34568733	CNTFR	13	0
Chr9	34903631	34903643	PHF24	13	1
Chr9	35397350	35397362	UNC13B	13	1
Chr9	35712736	35712748	TLN1	13	0
Chr9	91751310	91751322	ROR2	13	0
Chr9	94319990	94320002	NUTM2F	13	0
Chr9	116850562	116850574	ASTN2	13	0
Chr9	123557657	123557669	DENND1A	13	0
Chr9	127833973	127833985	ENG	13	0
Chr9	130127024	130127036	GPR107	13	0
Chr9	131674536	131674548	RAPGEF1	13	0
Chr16	56212024	56212039	GNAO1	16	0
Chr16	23487965	23487986	GGA2	20	1
Chr16	27927509	27927522	GSG1L	14	0
Chr16	48256192	48256205	LONP2	14	0
Chr16	67440611	67440624	ATP6V0D1	14	0
Chr16	70474275	70474288	FUK	14	0
Chr16	72998569	72998582	ZFHX3	14	0
Chr16	88583510	88583523	ZC3H18	14	0
Chr16	627239	627251	RAB40C	13	0
Chr16	12725679	12725691	CPPED1	13	0
Chr16	28171848	28171860	XPO6	13	1
Chr16	67781891	67781907	RBM10	16	0
Chr16	82867008	82867020	CDH13	13	0
Chr16	85360119	85360131	GSE1	13	1
Chr16	85554339	85554351	GSE1	13	0
Chr18	58213521	58213534	NEDD4L	14	0
Chr18	75283523	75283536	TSHZ1	14	0
Chr18	3706404	3706416	DLGAP1	13	0

Chr18	7092882	7092894	LAMA1	13	1
Chr18	48782818	48782830	CTIF	13	0
Chr18	52822357	52822369	DCC	13	1
Chr18	55411580	55411592	TCF4	13	0
Chr18	77044965	77044977	MBP	13	0
Chr18	79457068	79457080	NFATC1	13	0
Chr20	62682446	62682459	SLCO4A1	14	0
Chr20	963349	963361	RSPO4	13	1
Chr20	38337084	38337096	BPI	13	0
Chr20	63963986	63963998	ZNF512B	13	0
Chr1	19891480	19891493	OTUD3	14	0
Chr1	49468726	49468739	AGBL4	14	0
Chr1	184954774	184954787	FAM129B	14	0
Chr1	204345882	204345895	PLEKHA6	14	0
Chr1	244697186	244697199	DESI2	14	0
Chr1	1974728	1974740	CFAP74	13	1
Chr1	3842432	3842444	CEP104	13	0
Chr1	15743369	15743381	TMEM82	13	1
Chr1	19471468	19471480	CAPZB	13	1
Chr1	22880313	22880325	EPHB2	13	0
Chr1	26702713	26702725	ARID1A	13	0
Chr1	27965261	27965273	XKR8	13	1
Chr1	33811545	33811557	CSMD2	13	0
Chr1	39088469	39088481	MACF1	13	0
Chr1	40821447	40821459	KCNQ4	13	1
Chr1	45514727	45514739	PRDX1	13	0
Chr1	47223798	47223810	TAL1	13	1
Chr1	66142889	66142901	PDE4B	13	0
Chr1	66147487	66147499	PDE4B	13	1
Chr1	84860558	84860570	LPAR3	13	0
Chr1	95208508	95208520	TMEM56	13	1
Chr1	110603839	110603851	KCNA2	13	1
Chr1	115671267	115671279	VANGL1	13	0

Chr1	151430271	151430283	POGZ	13	1
Chr1	153420570	153420582	S100A8	13	0
Chr1	154541019	154541031	TDRD10	13	0
Chr1	158074004	158074016	KIRREL1	13	0
Chr1	160495112	160495124	SLAMF6	13	0
Chr1	163182329	163182341	RGS5	13	0
Chr1	165402990	165403002	RXRG	13	0
Chr1	167326841	167326853	POU2F1	13	1
Chr1	167878434	167878446	ADCY10	13	0
Chr1	172204373	172204385	DNM3	13	0
Chr1	197927202	197927214	LHX9	13	1
Chr1	200408162	200408178	ZNF281	16	1
Chr1	204346544	204346556	PLEKHG6	13	1
Chr1	206586049	206586061	RASSF5	13	0
Chr1	226377572	226377588	PARP1	16	0
Chr1	228367813	228367825	OBSCN	13	1
Chr1	230416470	230416482	PGBD5	13	0
Chr1	230669940	230669952	COG2	13	0
Chr1	231028564	231028580	FAM89A	16	0
Chr1	233339079	233339091	MAP3K21	13	0
Chr1	245796706	245796718	SMYD3	13	0
Chr1	246948024	246948036	ZNF695	13	0
Chr12	20621441	20621455	PDE3A	15	0
Chr12	32491014	32491028	FGD4	15	0
Chr12	527839	527851	B4GALNT3	13	0
Chr12	11854688	11854704	ETV6	16	0
Chr12	18313527	18313539	PIK3C2G	13	0
Chr12	21639031	21639043	LDHB	13	1
Chr12	44343413	44343425	TMEM117	13	0
Chr12	49296907	49296919	PRPH	13	0
Chr12	65894909	65894921	HMGA2	13	0
Chr12	68157994	68158006	IFNG	13	0
Chr12	71944370	71944382	TPH2	13	1

Chr12	79932543	79932555	PPP1R12A	13	1
Chr12	94267139	94267151	PLXNC1	13	1
Chr12	103717808	103717824	STAB2	16	0
Chr12	111803971	111803983	ALDH2	13	1
Chr12	119137158	119137170	SRRM4	13	0
Chr12	119786780	119786792	CIT	13	0
Chr12	120220876	120220888	PXN	13	1
Chr12	121750756	121750768	TMEM120B	13	0
Chr12	121751068	121751080	TMEM120B	13	0
Chr12	125118023	125118035	AACS	13	1
Chr12	128359709	128359721	TMEM132C	13	1
Chr12	130376121	130376133	PIWIL1	13	0
Chr13	32469334	32469348	N4BP2L2	15	0
Chr13	113312053	113312067	LAMP1	15	1
Chr13	23741664	23741676	MIPEP	13	0
Chr13	25796644	25796656	ATP8A2	13	0
Chr13	26150373	26150385	RNF6	13	0
Chr13	35896894	35896906	DCLK1	13	0
Chr13	41466141	41466153	RGCC	13	0
Chr13	42107779	42107791	DGKH	13	0
Chr13	43366706	43366718	ENOX1	13	0
Chr13	91633446	91633458	GPC5	13	0
Chr13	100511379	100511391	PCCA	13	1
Chr13	101170223	101170235	NALCN	13	1
Chr13	101281335	101281347	NALCN	13	0
Chr13	101361334	101361346	NALCN	13	0
Chr13	111256572	111256584	ARHGEF7	13	0
Chr13	113354230	113354242	GRTP1	13	1
Chr15	92444497	92444511	ST8SIA2	15	0
Chr15	29883117	29883130	TJP1	14	0
Chr15	34095366	34095379	EMC7	14	0
Chr15	36677828	36677841	C15orf41	14	0
Chr15	75473656	75473669	PTPN9	14	1

Chr15	23364196	23364212	GOLGA8S	16	0
Chr15	28388121	28388137	GOLGA8F	16	0
Chr15	28523499	28523515	GOLGA8G	16	0
Chr15	28702876	28702892	GOLGA8M	16	0
Chr15	30092561	30092577	GOLGA8J	16	0
Chr15	30404663	30404679	GOLGA8R	16	0
Chr15	30561562	30561578	GOLGA8Q	16	0
Chr15	30613623	30613639	GOLGA8H	16	0
Chr15	30801058	30801074	GOLGA8IP	16	0
Chr15	32393717	32393701	GOLGA8K	16	0
Chr15	32446029	32446045	GOLGA8O	16	0
Chr15	32603039	32603055	GOLGA8N	16	0
Chr15	32677708	32677720	SCG5	13	1
Chr15	34382371	34382387	GOLGA8A	16	0
Chr15	34528592	34528608	GOLGA7	16	0
Chr15	39994805	39994817	EIF2AK4	13	1
Chr15	40824938	40824950	PPP1R14D	13	0
Chr15	62659565	62659577	TLN2	13	0
Chr15	65062776	65062788	RASL12	13	0
Chr15	68331148	68331160	ITGA11	13	1
Chr15	68590837	68590849	CORO2B	13	0
Chr15	85118614	85118626	PDE8B	13	0
Chr15	88477695	88477707	MRPS11	13	0
Chr17	30027583	30027597	EFCAB5	15	1
Chr17	10185855	10185868	GAS7	14	0
Chr17	10725395	10725408	TMEM220	14	0
Chr17	28100556	28100569	NLK	14	0
Chr17	34124808	34124821	ASIC2	14	0
Chr17	56507513	56507526	ANKFN1	14	0
Chr17	81155028	81155041	AATK	14	1
Chr17	2212819	2212831	SMG6	13	0
Chr17	7411442	7411454	NLGN2	13	0
Chr17	17061394	17061406	MPRIP	13	0

Chr17	27609535	27609551	KSR1	16	0
Chr17	35696061	35696073	AP2B1	13	1
Chr17	37535977	37535989	SYNRG	13	1
Chr17	38806755	38806767	CWC25	13	1
Chr17	43526636	43526648	DHX8	13	0
Chr17	45130732	45130744	PLCG1	13	0
Chr17	67343440	67343452	PSMD12	13	0
Chr17	75736310	75736322	ITGB4	13	1
Chr17	78799571	78799583	USP36	13	1
Chr17	80433046	80433058	ENDOV	13	0
Chr17	81056672	81056684	BAIAP2	13	0
Chr17	82912579	82912591	TBCD	13	0
Chr19	10004764	10004778	COL5A3	15	0
Chr19	30595782	30595795	ZNF536	14	0
Chr19	35550820	35550833	ATP4A	14	0
Chr19	39872398	39872411	FCGBP	14	0
Chr19	50530504	50530517	LRRC4B	14	1
Chr19	10368278	10368290	TYK2	13	0
Chr19	11254911	11254923	DOCK6	13	0
Chr19	11723220	11723232	ZNF823	13	0
Chr19	13825433	13825445	ZSWIM4	13	0
Chr19	15678027	15678039	CYP4F12	13	0
Chr19	16096583	16096595	TPM4	13	1
Chr19	19132600	19132612	TMEM161A	13	0
Chr19	41362832	41362844	B9D2	13	1
Chr19	48479128	48479140	CYTH2	13	0
Chr19	50481536	50481548	EMC10	13	0
Chr19	51627824	51627836	SIGLEC5	13	1
Chr19	55135917	55135929	TNNT1	13	0
Chr19	58211657	58211669	ZNF274	13	1
Chr19	58285814	58285826	ZNF8	13	1
Chr21	40725762	40725774	DSCAM	13	0
Chr21	42386616	42386628	TMPRSS3	13	0

Chr21	42544478	42544494	SLC37A1	16	0
Chr21	43479441	43479453	HSF2BP	13	0
Chr21	43921919	43921931	AGPAT3	13	0
Chr22	32532887	32532901	SYN3	15	0
Chr22	19139654	19139666	ESS2	13	0
Chr22	23083969	23083981	RSPH14	13	1
Chr22	24242221	24242233	GGT5	13	0
Chr22	41175273	41175285	EP300	13	1
Chr22	44063730	44063742	PARVB	13	0
Chr22	47051991	47052003	TBC1D22A	13	0

Table S3. Transcript-specific primers used in qPCR.

Gene	Oligos	Primer sequence
**ATOH1**	F-primer	GTGCAGAAGCAGAGACGGCTA
	R-primer	GCTCGGACAAGGCGTTGATGTAG
**MASH1**	F-primer	GTCGGACGAGGGCTCTTACGA
	R-primer	TGCGATCACCCTGCTTCCAAAG
**ACTB**	F-primer	AGAGCTACGAGCTGCCTGACG
	R-primer	GGACTCCATGCCCAGGAAGGA
**VEGFA**	F-primer	CTCTACCTCCACCATGCCAAGTG
	R-primer	ACATCCATGAACTTCACCACTTCGTGA
**RNASEH1**	F-primer	CGTGAGCCACTGGATGGAGATG
	R-primer	ACGACGACGAAGTCTCCCATGTAG
**RNASEH2A**	F-primer	GGCGGGTCAAATACAACCTGAACTC
	R-primer	TGTCTCTGGCATCCCTACGGT
**RNASEH2B**	F-primer	CCCCTGTTCAGGAGAAGGAGC
	R-primer	CTGAAACTTCCCCTCCTTATCAGCCT
**RNASEH2C**	F-primer	GGGACTCGAAGTGTCGTTTCGG
	R-primer	GCTCCTCCTCTTGGTCGTCAGT
**TOP1**	F-primer	CGCTATCCTGAAGGCATCAAGTGG
	R-primer	CTGCTTTGGGGCTCAGCTTCA
**TOP2A**	F-primer	GTGACAGGTGGTCGAAATGGCT
	R-primer	AAGGGCTTGAGTTCCATCTCACCA
**TOP2B**	F-primer	TTGGCTGGCTCTGTTGCTGA
	R-primer	CCATGAAGCCGAGTTCCAAACTGAC
**TOP3A**	F-primer	CGAGCCCGATTCTCTGAGATCACA
	R-primer	CTCTGAAGCCGCAGGGTCT
**TOP3B**	F-primer	CGGCTCTACACGCAAGGCTAC
	R-primer	CTAACAACCGCTTCACCGTGTCG

Table S4. Specific primers used in quantification of locus topology.

Gene	Oligos	Primer sequence
**ATOH1**	F-primer	GTGCAGAAGCAGAGACGGCTA
	R-primer	GCTCGGACAAGGCGTTGATGTAG
**MASH1**	F-primer	GTCGGACGAGGGCTCTTACGA
	R-primer	TGCGATCACCCTGCTTCCAAAG
**MYOD1**	F-primer	CAAGCGCAAGACCACCAACG
	R-primer	CCGCTGGTTTGGATTGCTCG
