# Supplementary material for: Programmed genomic instability regulates neural transdifferentiation of human brain microvascular pericytes
Source: Genome Biol. 2021 Dec 9;22:334. doi: 10.1186/s13059-021-02555-0 (PMC8656028; doi:10.1186/s13059-021-02555-0)
Supplement: Supplementary file 2 — Additional file 2. Peer review history. [file 13059_2021_2555_MOESM2_ESM.docx]

**Review history**

**First round of review**

**Reviewer 1**

This paper explores some of the genomic mechanisms associated with the (by now well described) transdifferentiation phenomenon of microvascular pericytes into neurons. It proposes a nice hypothesis linking disruption of transcriptional programs to desuppression of neuronal lineage-related genes, including a potential role for DNA:RNA hybrids in stabilizing or promoting reprogramming of the gene expression program towards the neural transcriptome.

While the basic hypothesis is elegant, the general methodological rigor for some of the key experiments supporting this paper is weak and , in the opinion of this Reviewer at least, mandates additional studies to consolidate the proposed hypotheses:

For example, the authors claim 'global transcriptional arrest' but this is solely based on pharmacological disruption of nuclear actin filaments. There are other compounds available that directly target RNA polymerase II activity. It will be interesting to see if the observed transdifferentiation phenotype could be reproduced with non-actinergic drugs.

Likewise, the higher order chromatin topologies presented in figure 2 , which are based on bulk assays or microscopy, should be supplemented by unbiased Seq assays (ATAC-seq or Hi-C)

it is a bit unclear (at least to me)whether the transdifferentiation is stable (beyond the 3 day post-induction reported in one of their figures), what the % of pericytes is that convert into neural progenitor cells and what the fraction of progenitor cells is that can differentiate into neurons or glia.

**Reviewer 2**

This submission Rezaei-Lotfi et al used commercial available human microvascular pericyte as a culture model to establish a reprogramming protocol to induce neurogenesis using hypoxia combined with serum deprivation that repress mechano-transduction. Rezaei-Lotfi et al examined how underlying molecular mechanisms governing chromatin topology contribute to the reprograming process. They showed that transient global transcriptional arrest abrupted destabilization of the higher-order chromatin topology that chaperones lineage memory of pericytes. As a consequence, this leads within minutes to localized decompression of the repressed competing higher-order chromatin topology and expression of pro-neural genes. Transition to neural lineage is finally completed by induction of R-loops in key myogenic loci upon re-initiation of RNA polymerase activity, leading to depletion of the myogenic transcriptome and emergence of the neurogenic transcriptome. While it is novel to establish the link between chromatin topology with reprogramming process that requires repressed pericyte identity and initiate neurogenic identity, I do have some major concerns for this paper to publish at Genome Biology.
Major concerns:
1. To set up a TD model in culture using human pericytes, RT-PCR analysis of Mash1 and Atoh1 was not sufficient, ICH analysis and e-physic analysis of neurons derived from human pericytes should be assessed in long-term culture as well. This will confirm that the TD is sustainable for long-term culture.
2. In addition to cdk9 inhibitor, the authors should use genetic approach to knock-down cdk9 and observe whether it will show the same phenotype since the drug may have off-targets.
3. It is confusing for fig 3h. NeuN as a marker for mature neuron exhibiting in each image and nestin is used for the marker for pericytes or neural progenitors? nestin is known marker that recognize both pericytes and neural progenitors. Authors should clarify what all these markers for and give detailed explanations in results section. Also, all the markers in figure 3h should be examined in human pericyte population before neural induction. Quantification data should be displayed besides images.
4. Figure 4 should be better organized as friendly as possible for readers to follow and understand.
5. Global disruption of transcriptional landscape of pericytes should be replicated by transient removal of RNAP-II from human pericytes.
6. The authors named their TD model as endogenous nature TM using human pericytes. This is not an accurate terminology. It should be named genomic integration-free methodology to reprogram pericytes into neurons by altering environmental cues with hypoxia combined with serum starvation. This would be probably occurred following ischemic stroke in vivo, but not considered as a nature event in culture conditions.

**Authors’ response to reviewers**

**Reviewer 1**

We are grateful for the encouraging comments and constructive suggestions provided. We have endeavored to answer the questions and accommodate the suggestion by performing additional experiments as elaborated in the point-by-point rebuttal that follows.

*1. For example, the authors claim 'global transcriptional arrest' but this is solely based on pharmacological disruption of nuclear actin filaments. There are other compounds available that directly target RNA polymerase II activity. It will be interesting to see if the observed transdifferentiation phenotype could be reproduced with non-actinergic drugs.*

A: We thank the reviewer for this suggestion. In order to induce global synchronised transcriptional arrest, we required a compound with rapid onset of activity that inhibited RNAP-II in a reversible manner to mimic endogenous initial inhibition and subsequent activation of RNAP-II processivity. Various inhibitors of RNAP-II, such as alphaamanitin, were undesirable due to slow onset, irreversibility, or lack of selectivity. DRB, on the other, shows rapid onset, rapid (within minutes) reversibility, and high selectivity for CDK-9. As such, DRB-mediated inhibition of CDK-9 leads to inhibition of positive transcription elongation factor (P-TEFb) and CTD serine 2 dephosphorylation of RNAPII. In consequence, transcription elongation step is effectively inhibited. In the revised manuscript, we have employed multiple strategies to accommodates the suggestion of the reviewer.

•We have expanded the section on application of DRB by adding the following section as an introduction to the paragraph “In order to simulate F-actin-mediated suppression of RNAP-II and global transcriptional arrest, we required a compound with rapid onset of activity that inhibited RNAP-II in a reversible manner (42). Cyclin-dependent kinase9 (CDK-9) is a key component of the positive transcription elongation factor (P-TEFb). Inhibition of CDK-9 by application of 100 μM DRB (43), leads to dephosphorylation of CTD of RNAP-II and premature arrest of RNA polymerase-II, replicating global arrest of RNAP-II during Phase I of TD”, at page 7, paragraph 2, line 1 of the revised manuscript. In the former text, we have also added a new reference (ref: 42) that provides an overview of the rationale for selective application of inhibitors to target eukaryotic RNAP-II.

•In the revised manuscript, we have also employed a cocktail of two siRNAs in order to target CDK9 with higher specificity compared to DRB. The data presented as Figure 2f demonstrates that inhibition of CDK9 in growth medium (treatment I in the box plots of Figure 2f) enhances transcription from Atoh1 and Mash1 loci to the level that is comparable to transdifferentiating pericytes in neural induction medium (treatment II in the box plots of Figure 2f). Further, we now show that combination of RNAi-mediated inhibition of CDK9 and exposure to neural induction medium causes a synergistic amplification of transcription from Atoh1 and Mash1 loci (treatment III in the box plots of Figure 2f).

•Finally, we employed UVC-mediated abrupt stalling of RNAP-II as an additional method to investigate the role global transcriptional arrest in upregulation of transcription from pro-neural loci. The data presented in Fig. 2e provides further support for the role of transcriptional arrest in regulating the Phase I of transdifferentiation. The data can now be found at page 7, paragraph 2, line 14 of the revised document along with the new reference 44.

*2. Likewise, the higher order chromatin topologies presented in figure 2 , which are based on bulk assays or microscopy, should be supplemented by unbiased Seq assays (ATAC-seq or Hi-C).*

A: We thank the reviewer for this suggestion that has greatly improved the quality of our manuscript. In the revised manuscript, we employed ATAC-seq to investigate global chromatin accessibility of transdifferentiating pericytes. The data is presented as new Figure 3 of the revised manuscript in three sections. The global heatmap analysis, based on k-means algorithm, is presented in Fig. 3a which shows an abrupt global shift of chromatin accessibility relative to ENSEMBL-annotated transcription start site, in cluster 2 loci at t=2 min after induction of transdifferentiation. This finding corroborates findings regarding alteration of locus topology and RNA-seq, after induction of transdifferentiation. In the right column of the same figure, we present GO enrichment analysis of ATAC-seq results revealing that a genic cluster encoding cell cycle-related genes and cAMP signaling become inaccessible at t=2 min after transdifferentiation corroborating RNA-seq results obtained at t=5 min post-induction of transdifferentiation. Further, we show that loci encoding neuropeptide signaling (a major antagonistic pathway of cAMP signaling) and neural fate determination transcription factors become accessible after t=2 min post-induction of transdifferentiation. Finally we provide high-resolution mapping of ATAC-seq peaks to the genomic loci encoding MYOD1 and MASH1 which suggest increased accessibility of these two loci (more prominent in MASH1) at t=4 min, consistent with suggestion of transitioning into bistability and competition between promyogenic and pro-neurogenic fates. The relevant section in Results can now be found at page 8, paragraph 1, line 5 of the revised document.

*3. it is a bit unclear (at least to me) whether the transdifferentiation is stable (beyond the 3day post-induction reported in one of their figures), what the % of pericytes is that convert into neural progenitor cells and what the fraction of progenitor cells is that can differentiate into neurons or glia.*

A: Thank you for these interesting questions. In the following section, we have attempted to answer each question separately.

Regarding the stability and viability of cultured cells, it should be pointed out that both transdifferentiating and transdifferentiated pericytes are extremely resistant to apoptotic cues. We have now provided a new supplementary Fig. S1, which shows the outcome of transdifferentiation at t=14 days. We refer to this supplementary figure at page 4, paragraph 2, line 4 of the revised manuscript. While >95% of pericytes have completed neural transdifferentiation and express ß3-tubulin, one can only see rare accounts of extrachromosomal DNA (eDNA) and almost no apoptotic figures. Surprisingly, 90% of the population. This was also consistent with transcriptional profiling of pro-neural TFs fingerprinted at 5-min intervals and presented at Fig. 1c. The non-overlapping cycles were simulated by returning the transdifferentiating pericytes to GM after 20, 40, and 60 minutes to the growth medium to activate RTK and terminate transdifferentiation. However, given the possibility of overlapping cycles (i.e. re-initiation of the next round of transdifferentiation prior to completion of the 20-min cycle) and by integration of the transcriptional profile from Figure 1, transdifferentiation efficiency was estimated as 50% of population/20 min. In order to convert the population-level TD rate to transdifferentiation tendency (i.e. probability) of individual pericytes, we used Poisson binomial distribution to model TD with two key assumptions; that TD or resistance to TD is a binary event in the 20- min period and that TD of individual cells are independent from each other. Using this mathematical model, the conservative transdifferentiation tendency of individual pericytes is estimated as 40%/20min. Once again, if we assume that an ischemic period may rarely extend beyond 20min (e.g. a transient ischemic attack), the rate can be further simplified to the conservative baseline value of 40% for individual mural pericytes. This rate, however, can be programmed by various cues as follows.

Armed with knowledge that hypoxia and serum starvation alter the baseline expression of pro-neural transcription factors in growth medium (Figure 1), we questioned whether conditioning the pericytes by exposure to hypoxia and serum starvation alters the transdifferentiation tendency of these cells upon induction of transdifferentiation. The experiments using these exogenous cues revealed that while hypoxia alone reduces transdifferentiation propensity of pericytes, serum starvation enhances the tendency to transdifferentiate. This finding was again intuitively logical. One interpretation is that extrinsic hypoxia (i.e. hypermetabolic state) would trigger angiogenesis with minimal depletion of mural pericytes due to transdifferentiation. However, angiogenic remodeling ischemic vessels (i.e. vascular regression or pruning) could generate neural progenitor cells in anticipation of tissue damage (due to ischemia and unmet metabolic demand) leading to regeneration of the damaged tissue. Further to exogenous cues, we reasoned that accumulation of genomic damage, and the resultant genomic instability could tip the balance in favor of transdifferentiation (one scenario could be aging-related genomic instability). We employed short pulses of UV irradiation prior to transdifferentiation which revealed that enhanced transdifferentiation tendency subsequent to genomic instability. These endogenous cues alter the transdifferentiation tendency of pericytes by 2-fold as referenced to the transdifferentiation rate observed in culture. This discussion can now be found under new subheading “TD propensity of pericytes is programmed by prior exposure to stressors” at page 13 of the revised document.

Finally, the new supplementary Fig. S1 shows the sublineage transdifferentiation of pericyte to GFAP+/GS+ glial cells in vitro. To this end, we have now added new discussion under the same subheading as above regarding potential programmability of sublineage differentiation outcome of pericytes. We provide the example of commitment to glial differentiation that requires input from Notch-1 signaling pathway (ref: 65). This pathway, on the other hand, not only is regulated by history of exposure to stressors but it is also records and carries the memory of exposure to subsequent cellular generations (ref: 66). We, therefore, propose that the programmability of neural TD by stressors provides an effective path for guided regeneration of damaged neural tissues based on archived but accessible memory of previous exposure to stressors.

**Reviewer 2**

We would like to thank the reviewer for the constructive suggestions provided. In the revised manuscript, we have endeavored to accommodate these suggestions that have improved the scientific quality of our manuscript. Following is a point-by-point rebuttal that frames how the suggestions have been accommodated in the revised document.

*1. To set up a TD model in culture using human pericytes, RT-PCR analysis of Mash1 and Atoh1 was not sufficient, ICH analysis and e-physic analysis of neurons derived from human pericytes should be assessed in long-term culture as well. This will confirm that the TD is sustainable for long-term culture.*

A: Thank you for the comment provided. In the revised document, we have added a new supplementary Fig. S1 which shows the outcome of transdifferentiation at t=14 days. While >95% of pericytes have completed neural transdifferentiation and express ß3-tubulin, one can only see rare accounts of extrachromosomal DNA (eDNA) and almost no apoptotic figures. Surprisingly, <5% of transdifferentiation-resistant pericytes (expressing alpha-smooth muscle actin), remain viable at neural induction medium which is depleted of any growth factors including FGF-2. From a teleological perspective, this finding is intuitively logical as exhaustion by apoptosis of transdifferentiating cells would have been catastrophic due to depletion of mural pericytes and subsequent micro-aneurysmal changes of terminal blood vessels. One may argue that the latter observation reflects developmental origin of pericytes. As detailed in elegant studies by Etchevers et al (ref:19), neural pericytes originate from a population of migrating neural crest cells. Hence, it can be argued that these cells had embarked upon neural differentiation journey when they were captured by microvasculature as quasi-stable (metastable) cells. We would also like to point out that in elegant studies by Karow et al (ref 4 of the revised document), evidence electrophysiological evidence is provided regarding functionality of neurons generated from transdifferentiating pericytes. It is also noteworthy that in previous published work, we have provided in vivo evidence that interneurons generated from transdifferentiating pericytes, not only survive but also integrate into local neuronal networks.

*2. In addition to cdk9 inhibitor, the authors should use genetic approach to knockdown cdk9 and observe whether it will show the same phenotype since the drug may have off-targets.*

A: We are grateful for this suggestion. In the revised document, we have used a cocktail of two siRNAs in order to target CDK9. The data presented as Figure 2f demonstrates that inhibition of CDK9 in growth medium (treatment I in the box plots of Figure 2f) enhances transcription from Atoh1 and Mash1 loci to the level that is comparable to transdifferentiating pericytes in neural induction medium (treatment II in the box plots of Figure 2f). Further, we now show that combination of RNAi-mediated inhibition of CDK9 and exposure to neural induction medium causes a synergistic amplification of transcription from Atoh1 and Mash1 loci (treatment III in the box plots of Figure 2f).

*3. It is confusing for fig 3h. NeuN as a marker for mature neuron exhibiting in each image and nestin is used for the marker for pericytes or neural progenitors? nestin is known marker that recognize both pericytes and neural progenitors. Authors should clarify what all these markers for and give detailed explanations in results section.*

A: We agree that the rationale for choosing various markers needs to be better explained in the results section. As such, we have added the following statement to page 10, last sentence of the revised document “We employed Nestin immunoreactivity to identify cells which become committed to differentiation and loose stemness. NeuN was used as a reporter of neural lineage reprogramming. Accordingly, Nestin+/NeuN+ immunoreactivity characterised reprogrammed undifferentiation cells as opposed to Nestin+/NeuN- immunoreactivity of original pericytes”.

Also, all the markers in figure 3h should be examined in human pericyte population before neural induction.

A: We apologize for this oversight. We have now provided this information as a new panel in Supplementary Fig. S4 of the revised document.

Quantification data should be displayed besides images.

A: In agreement with the comment provided, we have generated a new panel that contains the requested information. However, in order to avoid overcrowding the panel, we have added the data to supplementary Fig. S4 of the revised document.

*4. Figure 4 should be better organized as friendly as possible for readers to follow and understand.*

A: We agree that the original arrangement of Figure 4 made it difficult to access it. To accommodate this suggestion, we have now rearranged the figure in the revised document to make it more accessible and user-friendly. To this end, the left column demonstrates GO enrichment analysis of missing transcripts. The right column, on the other hand, is not dedicated to the analysis of remaining transcriptomic profile and its remodeling. Please note that Figure 4 of the original document is now renumbered as Figure 5 owing to addition of a new Figure 3. Please also not that we had to remove the Sankey diagram of original Figure 4 (schematic summary diagram) to create space for re-arrangement of the figure.

*5. Global disruption of transcriptional landscape of pericytes should be replicated by transient removal of RNAP-II from human pericytes.*

A: We thank the reviewer for this suggestion. In order to induce global synchronised transcriptional arrest, we required a compound with rapid onset of activity that inhibited RNAP-II in a reversible manner to mimic endogenous initial inhibition and subsequent activation of RNAP-II processivity. Various inhibitors of RNAP-II, such as alphaamanitin, were undesirable due to slow onset, irreversibility, or lack of selectivity. DRB, on the other, shows rapid onset, rapid (within minutes) reversibility, and high selectivity for CDK-9. As such, DRB-mediated inhibition of CDK-9 leads to inhibition of positive transcription elongation factor (P-TEFb) and CTD serine 2 dephosphorylation of RNAPII. In consequence, transcription elongation step is effectively inhibited. In the revised manuscript, we have employed multiple strategies to accommodates the suggestion of the reviewer.

•We have expanded the section on application of DRB by adding the following section as an introduction to the paragraph “In order to simulate F-actin-mediated suppression of RNAP-II and global transcriptional arrest, we required a compound with rapid onset of activity that inhibited RNAP-II in a reversible manner (42). Cyclin-dependent kinase9 (CDK-9) is a key component of the positive transcription elongation factor (P-TEFb). Inhibition of CDK-9 by application of 100 μM DRB (43), leads to dephosphorylation of CTD of RNAP-II and premature arrest of RNA polymerase-II, replicating global arrest of RNAP-II during Phase I of TD”, at page 7, paragraph 2, line 1 of the revised manuscript. In the former text, we have also added a new reference (ref: 42) that provides an overview of the rationale for selective application of inhibitors to target eukaryotic RNAP-II.

•As pointed out earlier, we have also employed a cocktail of two siRNAs in order to target CDK9 with higher specificity compared to DRB (Figure 2f).

•Finally, we employed UVC-mediated abrupt stalling of RNAP-II as an additional method to investigate the role global transcriptional arrest in upregulation of transcription from pro-neural loci. The data presented in Fig. 2e provides further support for the role of transcriptional arrest in regulating the Phase I of transdifferentiation. The data can now be found at page 7, paragraph 2, line 14 of the revised document along with the new reference 44.

*6. The authors named their TD model as endogenous nature TM using human pericytes. This is not an accurate terminology. It should be named genomic integrationfree methodology to reprogram pericytes into neurons by altering environmental cues with hypoxia combined with serum starvation. This would be probably occurred following ischemic stroke in vivo, but not considered as a nature event in culture conditions.*

A: We agree with reviewer that the adopted terminology can be improved to reflect the utilized methodology more accurately. To accommodate this suggestion, we have made the following changes to the manuscript:

•We have renamed natural TD as integration-free TD at page 3, paragraph 1, line 8 of the revised manuscript.

•We have also added three references (references 15-17) to establish direct link to the literature. We thank the reviewers again for providing this opportunity to improve the document.

**Second round of review**

**Reviewer 1**

The authors have properly responded to previous review with additional experiments and all in all, the manuscript is now much improved and I have no additional comments.

**Reviewer 2**

The authors have addressed most of my concerns. just one minor thing regarding use of NESTIN+/NeuN+ and Nestin+/NeuN- to characterize the cell populations. NeuN is a maker to label fully mature post-mitotic neurons. If the purpose of the markers is to characterize reprogrammed undifferentiation cells, the authors should better use B3-tubulin as early-born neuron marker, but not NeuN which a fully-mature neuron marker.

**Authors’ response to reviewers**

**Reviewer 2**

We do apologize for this oversight in not responding to this question. Following is the rationale for choice of the markers and the strategies employed to accommodate this concern.

1. *The authors have addressed most of my concerns. just one minor thing regarding use of NESTIN+/NeuN+ and Nestin+/NeuN- to characterize the cell populations. NeuN is a maker to label fully mature post-mitotic neurons. If the purpose of the markers is to characterize reprogrammed undifferentiation cells, the authors should better use B3- tubulin as early-born neuron marker, but not NeuN which a fully-mature neuron marker.*

A: Thank you for raising this important point. In agreement with this comment, we have rephrased the sentence regarding Nestin immunoreactivity (page 11 sentence 1 of the revised document) to avoid any ambiguity. The revised statement maintains “We employed Nestin immunoreactivity to identify cells which become Nestin- upon committing to differentiation and loss of stemness”. We have also added a new reference to support this statement (ref. 51). In agreement with the reviewer’s suggestion, we have now added a new statement regarding the application of NeuN and β3-tubulin as neuronal markers which maintains that “NeuN and β3-tubulin (Tuj-1), on the other hand, were used as specific markers of mature and immature neurons, respectively”. The latter statement is supported by two new reference (refs. 52, 53). We have now corrected an error in the following sentence which now reads as “Accordingly, Nestin+/NeuN+ immunoreactivity characterised reprogrammed differentiated cells as opposed to Nestin+/NeuN- immunoreactivity of original pericytes”.
